# Supplementary material for: Distinct roles of Atf3, Zfp711 and Bcl6b in early embryonic hematopoietic and endothelial lineage specification
Source: Development. 2025 Dec 8;152(23):dev204792. doi: 10.1242/dev.204792 (PMC12746078; doi:10.1242/dev.204792)
Supplement: Supplementary information [file develop-152-204792-s1.pdf]

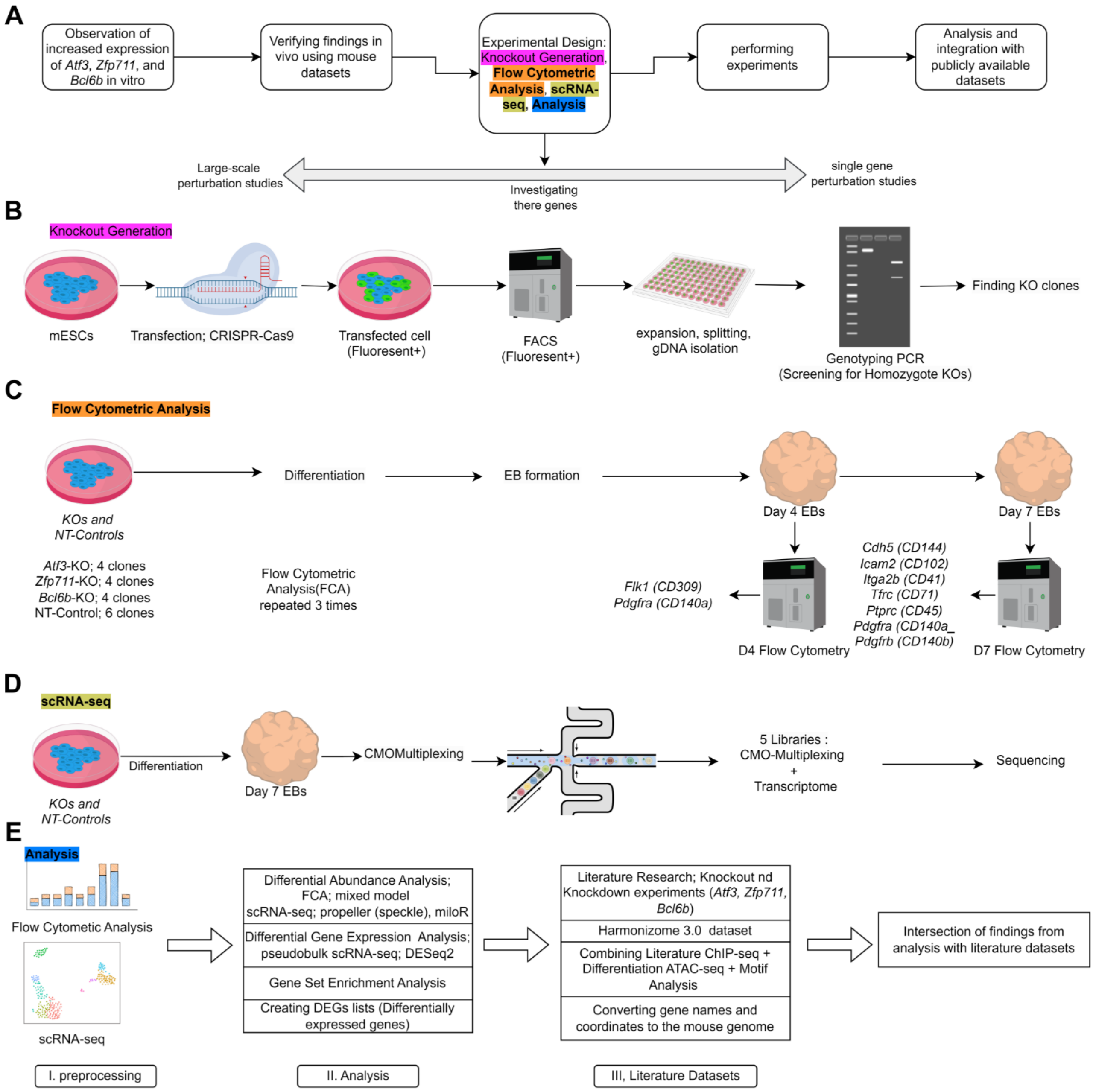

Fig. S1. Steps of the study, and details of the individual steps. (A) Shows the overall timeline of the study. (B) the knockout cell line generation steps. (C) the Flow Cytometric Analysis workflow. (D) the multiplexed scRNA-seq workflow. (E) the analysis workflow of the FCA and scRNA-seq datasets.

Development • Supplementary information

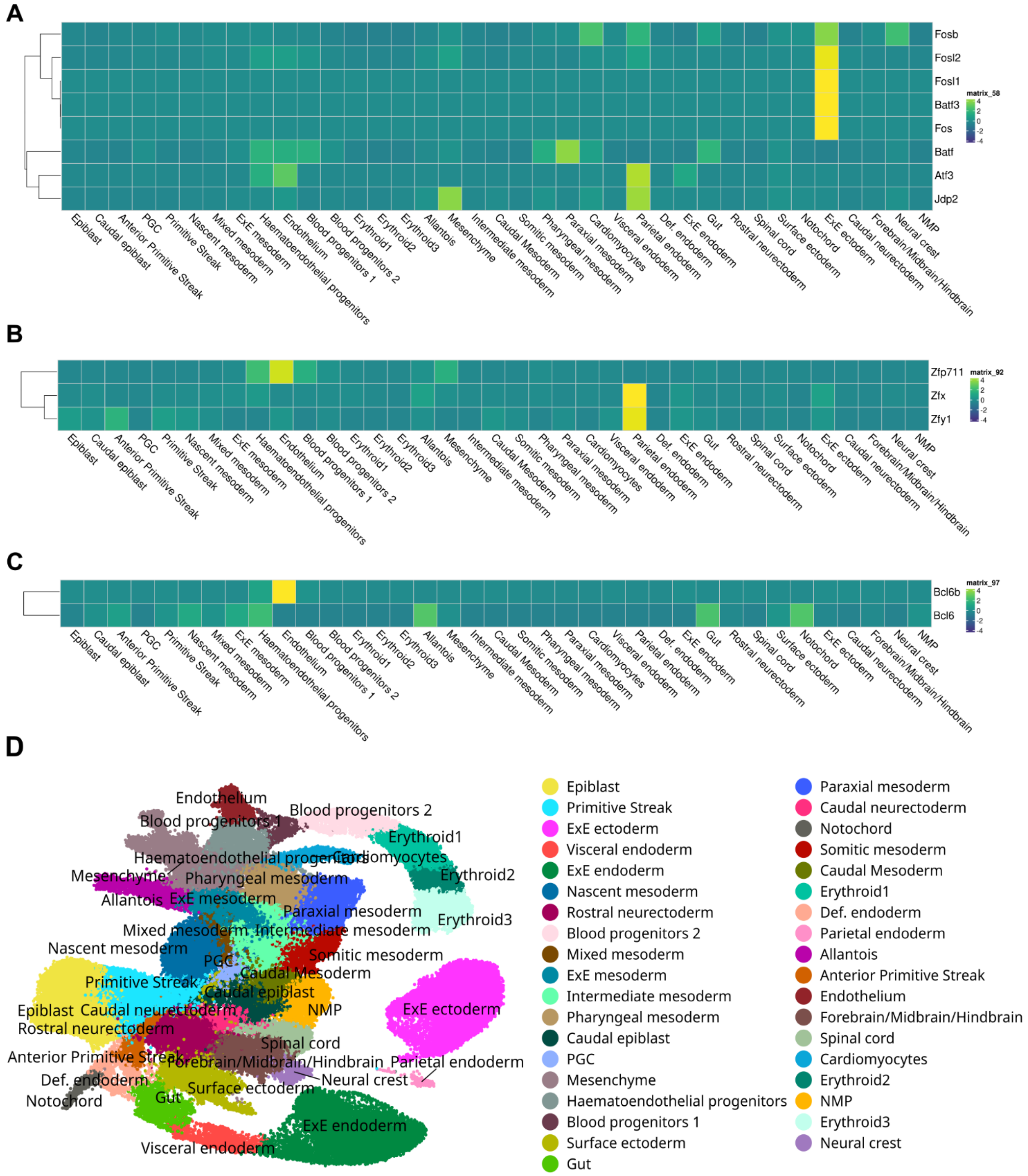

Fig. S2. Selected TFs and the expression of their paralog(s) *in vivo*. (A) shows the expression of *Atf3* and its paralogs, as documented in the mouse gastrulation atlas. (B) the expression of *Zfp711* and its paralogs (*Zfy2* shows no expression and was removed). (C) the expression of *Bcl6b* and its paralog. (D) the annotation of the cell types in UMAP from the mouse gastrulation atlas (Pijuan-Sala et al., 2019).

Development • Supplementary information

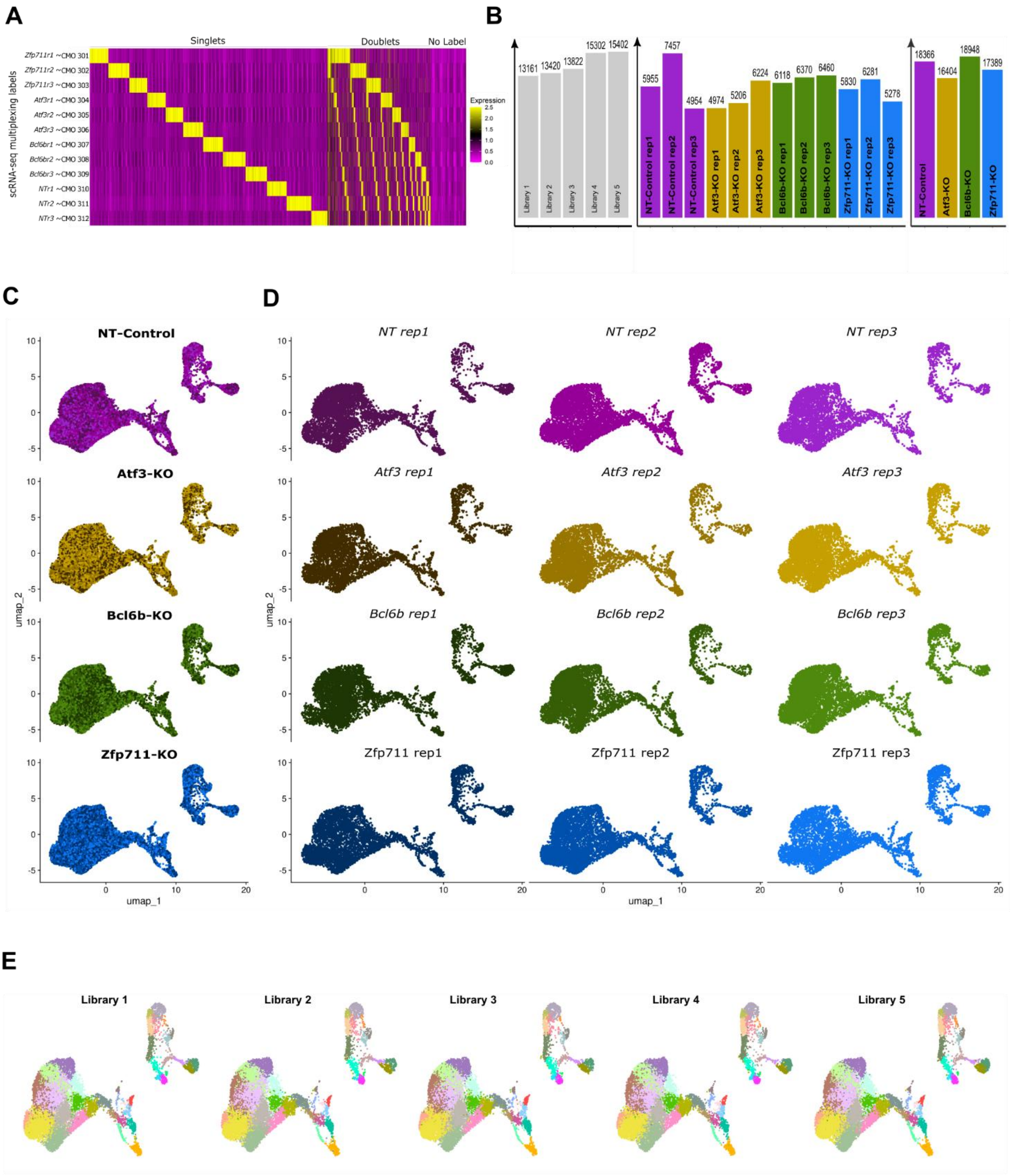

Fig. S3. Demultiplexing scRNA-seq and cell distribution. (A) An example of demultiplexing, using the Seurat-HTODemux function for a library. The Y-axis shows the samples and CMO labels. The X-axis shows individual cells. The heatmap shows CMO label counts and categorizes cells into singlets, doublets, and negative/No Signal/unassigned. (B) Distribution of number of cells after quality control. The panel on the left shows the number of cells in each library. The panel in the middle shows the number of cells from each sample/clone. The panel on the right shows the number of cells for each experimental condition. (C) UMAPs are split by conditions. (D) UMAPs are split by samples. (E) UMAPs are split by lanes. Different colors represent different sub-clusters.

Development • Supplementary information

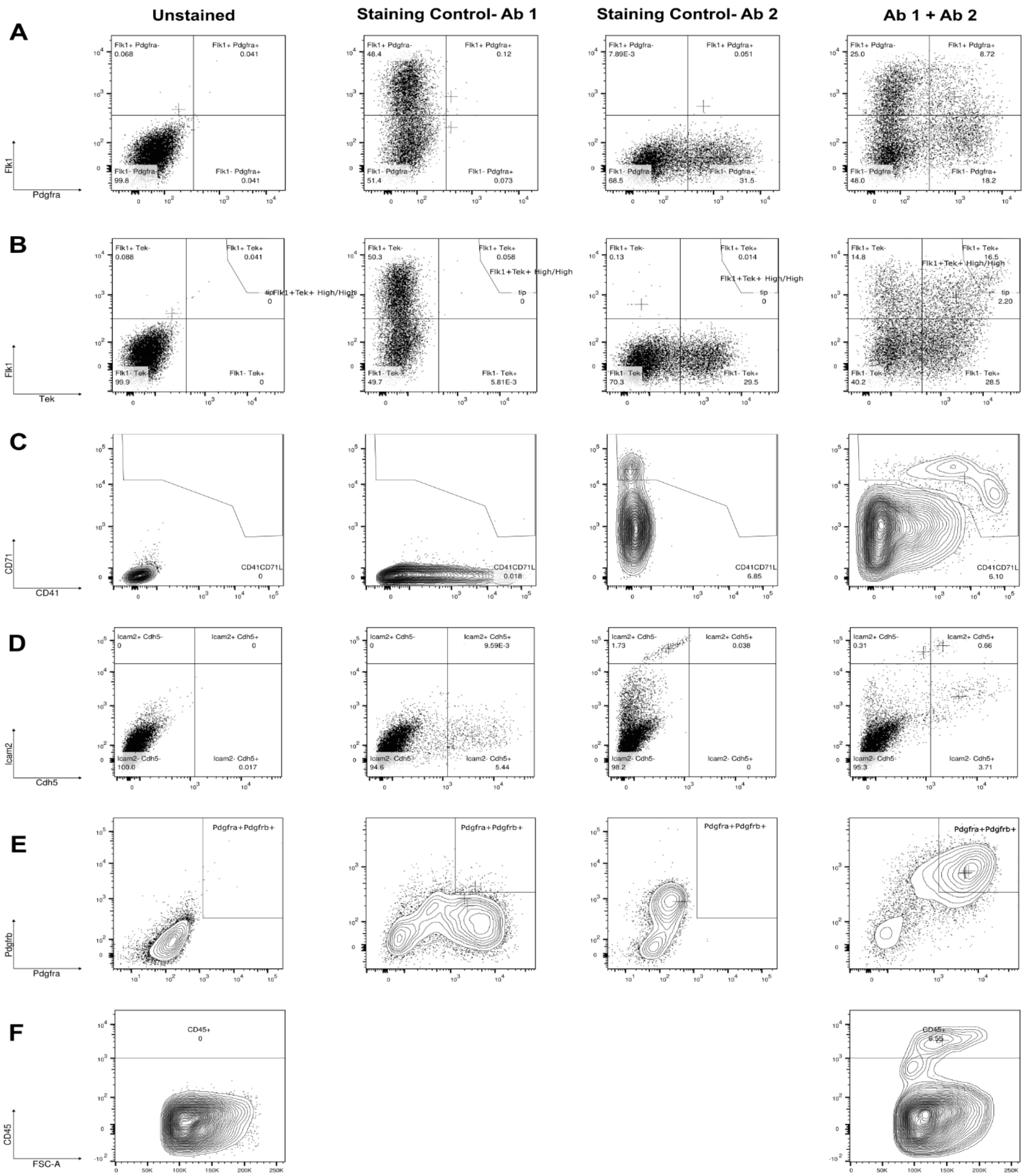

Fig. S4. Staining for cell surface markers and example flow cytometry gating. (A) Flk1/Pdgfra staining on day 4 (D4). (B) Flk1/Tek staining on D4. (C) CD41/CD71 staining on D7. (D) Icam2/Cdh5 on D7. (E) Pdgfra/Pdgfrb on D7. (F) CD45 staining on D7.

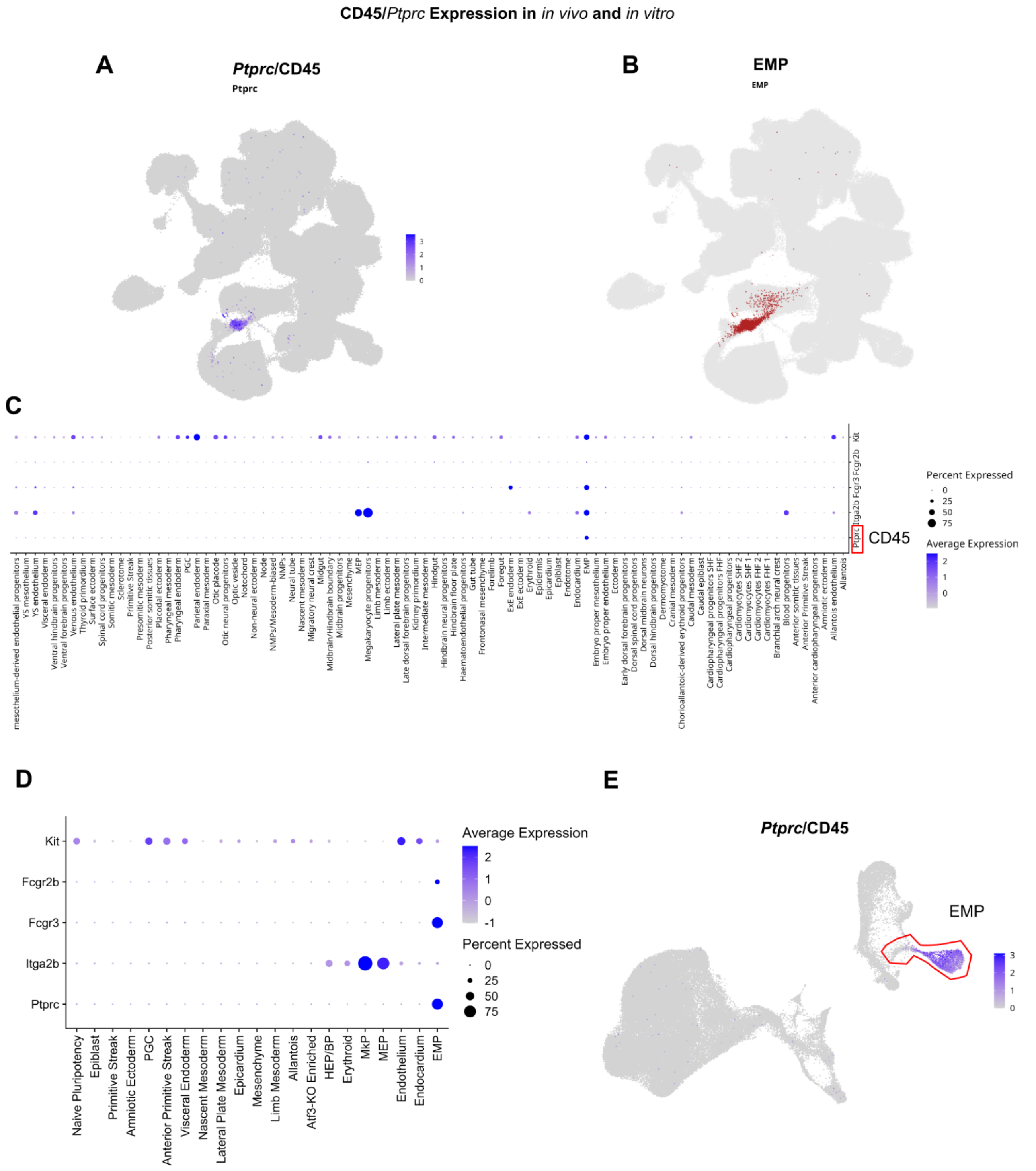

Fig. S5. CD45/Ptprc expression in *in vivo* and *in vitro* single-cell datasets. (A) UMAP of the *in vivo* reference atlas colored by *Ptprc* (CD45) expression; color intensity reflects scaled average expression (bar at right). (B) Location of annotated erythro-myeloid progenitors (EMP) on the same UMAP (red points). (C) Dot plot showing *Ptprc* expression across atlas cell types; dot size indicates the percentage of cells expressing the gene and color indicates average expression. *Ptprc* is highly enriched in EMP-related populations and largely absent in other populations. (D) Dot plot for selected hematopoietic markers (*Kit, Fcgr2b, Fcgr3, Itga2b,* and *Ptprc*) across major *in vitro* differentiation groups, demonstrating co-expression of CD45 with EMP/hematopoietic states. (E) UMAP of the *in vitro* dataset colored by *Ptprc* expression with the EMP-like region outlined in red. In all dot plots, larger dots denote higher percent expressed, and deeper blue denotes higher average expression.

Development • Supplementary information

1	2	3	4	5	6	7	8	9	10	11	12	13	14	15	16	17	18	19	20	21	22	23
Groups	Clusters	Subclusters	Ident.	Details from mapping to the *in vivo* atlas	Positive/Marker Genes	Freq.	Cell#	C.Cycle	Freq. Cell Cycle			Freq. Conditions				Cell no. Coditions				Expression		
									G1	S	G2M	Control	ΔAtf3	ΔZfp711	ΔBcl6b	Control	ΔAtf3	ΔZfp711	ΔBcl6b	*Atf3*	*Zfp711*	*Bcl6b*
Early Differentiation	Naive-Pluripotency	Naive-Pluripotency	*Zfp42*	Pooled;	*Morc1, Ooep, Sox2, Esrrb, Klf2, Zfp42*	1,6	1135	S-G2M	3,1	60,8	36,1	1,6	2,0	1,4	1,4	299	320	246	270	0,27	0,07	0,00
	Epiblast	Epiblast	*Pou5f1*	Pooled;	*Pim2, Tcfl5, Kcnj3, Pou3f1, Nanog, Pou5f1*	1,4	982	S-G2M	2,2	57,3	40,4	1,6	1,6	1,6	0,8	294	260	272	156	1,18	0,06	0,03
	Primitive-Streak	Primitive-Streak#1	*T*	Pooled;	*T, Igfbp3, Ppp4r4, Appl1, Rab18, Fgf8*	0,8	557	S-G2M	16,3	39,5	44,2	0,8	0,8	1,0	0,5	149	135	170	103	1,29	0,04	0,01
		Primitive-Streak#2		Pooled;	*Mesp1, Limch1, Wnt5a*	0,7	503	S-G2M	16,5	46,9	36,6	0,8	0,6	0,9	0,5	142	106	151	104	2,04	0,02	0,03
	Amniotic-Ectoderm	Amniotic-Ectoderm#1	*Wnt4*	Pooled, EP, PosS;	*Sfn, Cxcl16, Synpo2l, Ltb, Dusp14, Nhsl1*	0,6	445	S-G2M	13,9	39,8	46,3	0,8	0,6	0,7	0,5	146	93	115	91	2,50	0,01	0,00
		Amniotic-Ectoderm#2		Pooled, EP, PosS;	*Cldn3, Tacstd2, Wnt4, Krt7*	0,7	500	S-G2M	22,8	26,6	50,6	0,8	0,4	0,6	0,9	146	65	113	176	1,55	0,14	0,00
		Amniotic-Ectoderm#3		Pooled, EP, PosS;	*Anxa1, Cd44, Robo2, Cdkn2b, Zeb2*	0,2	120	G1-S-G2M	49,2	23,3	27,5	0,2	0,1	0,2	0,2	28	19	29	44	0,27	0,03	0,01
	PGC	PGC	*Prdm1*	Pooled;	*Ttc28, Dnd1, Hmcn1, Prdm1*	0,7	474	S-G2M	15,0	49,8	35,2	0,7	0,7	0,8	0,5	127	107	145	95	0,69	0,04	0,00
	Visceral-Endoderm	Anterior-Primitive-Streak	*Foxa2*	Pooled;	*Fgf5, Fgf10, Lhx1, Gsc, Sox17, Dgkk, Otx2,*	0,2	171	S-G2M	21,1	47,4	31,6	0,3	0,2	0,3	0,2	47	36	54	34	0,34	0,02	0,02
		Visceral-Endoderm		Pooled, MedS;	*Ttr, Spink1, Ctsh, Clic6, Dpp4, S100g, Car4*	0,3	223	G1-S-G2M	48,0	25,6	26,5	0,3	0,2	0,5	0,3	50	38	83	52	1,95	0,45	0,00
	Nascent-Mesoderm	Nascent-Mesoderm	*Eomes*	Pooled;	*Mixl1, Eomes, Wnt3, Tbx3, Rftn1, Grrp1*	2,2	1577	S-G2M	11,9	50,9	37,2	2,5	2,3	2,2	1,8	460	385	385	347	0,36	0,06	0,00
Late Mesoderm	Mesenchyme-Epicardium (Hoxb+)	Lateral-Plate-Mesoderm	*Msx2*	Pooled, PosS, EP;	*Fgf15, Tnnt3, Msx2, Foxo4, Ms4a4d*	2,7	1947	S-G2M	11,1	54,2	34,7	3,0	2,7	3,2	2,1	548	437	555	407	0,41	0,35	0,00
		Epicardium#1	*Nfib, Lhx2*	MedS, EP, PosS;	*Cacna1d, Htra1, Adgrg6, Twist1, Col13a1*	3,4	2435	G1-S-G2M	44,1	26,5	29,4	4,1	2,7	3,8	3,0	757	445	664	569	1,49	0,41	0,00
		Epicardium#2		MedS, EP;	*Nrxn3, Nfib, Fmn1, Tmod1, Lhx2*	1,7	1239	G1-S-G2M	50,5	18,4	31,1	2,1	1,7	1,9	1,3	389	275	323	252	1,46	0,37	0,00
		Epicardium#3		MedS, PosS, EP;	*Plxna4, Epha3, Plcl1, Nrxn3*	14,1	10050	G1-s-g2m	59,2	15,3	25,5	13,1	14,3	17,0	12,4	2400	2344	2950	2356	3,02	0,37	0,00
		Mesenchyme#1	*Ezr, Bnc1*	EP, MedS;	*Stard8, Slc2a1, Cobll1, Bmp4*	3,5	2454	S-G2M	20,7	42,1	37,2	3,2	3,5	5,1	2,2	584	572	887	411	0,77	1,01	0,00
		Mesenchyme#2		EP, MedS, PosS;	*Upk3b, Podxl, Cryab, Wisp1, Ak5, Ezr, Tnni1*	8,2	5853	S-G2M	15,6	39,5	44,9	7,6	8,8	10,3	6,4	1404	1446	1788	1215	1,42	0,24	0,00
	Limb-Mesoderm-Allantois (Hoxa+)	Limb-Mesoderm#1	*Prrx1, Mecom*	PS, EP;	*Greb1, Lef1, Cdx2, Isl1*	2,6	1836	S-G2M	16,9	45,0	38,0	2,7	2,1	3,1	2,5	488	337	543	468	1,10	0,30	0,00
		Limb-Mesoderm#2		EP, MedS;	*Dach1, Prrx1, Mecom, Ebf1, Crabp2, Foxp2*	4,7	3351	S-G2M	24,2	39,5	36,3	5,3	4,6	3,6	5,2	980	762	632	977	1,26	0,35	0,00
		Allantois#1	*Pitx1, Tbx4*	PosS, EP, MedS;	*Actc1, Krt19, Upk3b, Myl9, Bmp4, Frzb*	7,3	5179	S-G2M	13,2	42,6	44,2	7,4	7,7	5,0	8,9	1357	1265	864	1693	1,38	0,28	0,00
		Allantois#2		PosS, EP, MedS;	*Wnt2, Ccser1, Rbfox1, Fas, Scube1, Fgf12*	11,7	8355	G1-s-g2m	61,9	9,4	28,7	10,7	12,2	8,6	15,2	1973	1997	1496	2889	3,82	0,27	0,00
		Allantois#3		PosS, EP, MedS;	*Fgf12, Ccser1, Scube1, Wnt2, Negr1*	8,8	6286	S-G2M	3,9	50,2	45,9	8,6	10,0	6,2	10,5	1588	1638	1070	1990	2,34	0,28	0,00
		Allantois#4		PosS, EP, MedS;	*Igfbp7, Sparcl1, Cdo1, Wnt2, Cntn4, Tbx4,*	8,2	5863	G1-s-g2m	62,5	17,4	20,1	9,0	7,7	6,4	9,6	1662	1266	1114	1821	2,58	0,24	0,00
	ΔAtf3- Enriched	*ΔAtf3* -Enriched	*Ifit1*		*Ifit1, Isg15, Gbp7, Ifit3, Usp18, Rsad2*	0,3	202	G1-S-G2M	43,6	23,8	32,7	0,1	0,9	0,1	0,1	13	154	19	16	3,93	0,27	0,00
Hematopoietic	HEP/BP	HEP/BP	*Tal1*	Pooled, YS;	*Tal1, Lmo2, Runx1, Slc39a8, Fgf3, Myb,*	1,4	1025	S-G2M	3,8	53,9	42,3	1,2	1,5	2,1	1,1	218	243	364	200	0,74	0,27	0,00
	Erythroid	Erythroid#1	*Cited4*	YS;	*Glrx5, Tspan33, Nt5dc2, Mthfd2, Asns*	1,4	971	S-G2M	2,7	39,9	57,5	1,2	1,4	1,8	1,2	212	229	306	224	0,59	0,07	0,00
		Erythroid#2		YS;	*Tpx2, Cpox, Cited4, Ssx2ip, Cenpk*	0,7	502	S-G2M	1,0	28,5	70,5	0,7	0,6	1,0	0,6	125	94	173	110	1,17	0,18	0,01
		Erythroid#3		YS;	*Slc4a1, Ermap, Hemgn, Rhag, Acp5, Kel*	0,7	487	S-G2M	1,8	25,7	72,5	0,7	0,6	0,8	0,6	127	104	144	112	1,38	0,11	0,00
		Erythroid#4		YS;	*Hbb-y, Hba-a2, Alas2, Slc25a21, Gypa*	2	1438	G1	96,0	1,5	2,5	1,8	2,0	2,5	1,9	324	334	428	352	2,44	0,08	0,00
	Megakaryocyte Progenitors (MkP)	MkP#1	*Plek*	YS;	*Rab27b, Gp1bb, Gp9, Treml1, Sla2*	0,5	345	S-G2M	20,9	32,5	46,7	0,4	0,4	0,6	0,5	80	67	105	93	0,35	0,08	0,00
		MkP#2		YS;	*Thbs1, Gp1bb, Gp9, Treml1, Alox12, Vwf*	0,6	407	G1	82,1	2,5	15,5	0,5	0,4	0,7	0,7	91	71	119	126	0,37	0,03	0,00
		MEP	*Trib3*	YS;	*Hsd3b1, Thbs1, Gp1bb, Gp5, Tubb1*	0,1	106	G1	88,7	1,9	9,4	0,1	0,1	0,2	0,2	21	24	30	31	2,19	0,30	0,00
	Endothelium	Endothelium#1	*Fli1, Erg*	EP, Pooled;	*Gadd45g, Ramp2, Mest, Sparc, Col4a1*	1,1	807	G1-S-G2M	31,6	34,0	34,4	1,3	0,9	1,4	1,0	237	144	235	191	2,85	0,75	0,20
		Endothelium#2		EP, AntS, EP;	*Cdh5, Cldn5, Ldb2, Emcn, Col4a1, Ecscr*	1	731	G1-s-g2m	56,2	16,8	26,9	1,2	0,8	1,1	1,0	228	124	186	193	3,21	1,41	0,62
		Endothelium#3		YS;	*Rsad2, Ifit3, Ifit3b, Iigp1, Gbp4, Ifit1*	0,1	54	G1-s-g2m	53,7	24,1	22,2	0,0	0,2	0,1	0,0	9	26	10	9	2,06	2,02	0,64
		Endocardium	*Eng*	EP;	*Cyp26b1, Pdlim3, Ccdc80, Mal, Il1r1*	0,3	199	G1-s-g2m	55,3	15,6	29,1	0,3	0,4	0,2	0,3	49	60	42	48	5,76	0,58	0,38
	Erythro-Myeloid Progenitors (EMP)	EMP#1	*Spi1*	YS;	*Nrg1, Alox5ap, Gm4779, Adgrg3, Cd34*	0,6	427	G1-S-G2M	25,5	38,6	35,8	0,6	0,7	0,5	0,7	107	107	88	125	0,52	0,75	0,02
		EMP#2		YS;	*Il17ra, Cd52, Cacnb2, Ramp1, Prtn3,*	0,4	309	G1-S-G2M	45,3	25,6	29,1	0,4	0,4	0,5	0,4	77	69	80	83	3,65	0,42	0,00
		EMP#3		YS;	*Ccl4, Ccl3, Lpl, Apbb1ip*	1	738	S-G2M	17,6	44,4	37,9	1,0	0,7	1,3	1,1	185	120	230	203	5,97	0,10	0,00
		EMP#4		YS;	*Sepp1, C1qa, Fcgr3, Lyz2, Ms4a6b,*	1,2	824	G1	69,9	11,4	18,7	1,3	0,5	1,0	1,6	245	86	181	312	10,07	0,09	0,01

Fig. S6. Details of cell lineage, as group, cluster, and sub-cluster, nomenclature, and characteristics of the cell populations. Columns 1-3 show the annotation levels; 4 the genes used for identification/annotation of the sub-clusters; 5 the additional details about mapped anatomic location in *in vivo* ; 6 the marker genes for each sub-cluster; 7 the frequency of the sub-clusters; 8 the number of cells; 9 dominant cell cycle stage(s); 10-12 the frequency of the cell cycle stages (low [white] → high [green]); 13-16 the frequency of each condition in each sub-cluster (low [yellow] → high [green]); and 17-20 the number of the cells from each condition. 21-23 average expression of TFs *Atf3, Zfp711, and Bcl6b* in each sub-cluster, expression level is colored highlighted with low (light) → high (dark) tones of orange, blue, and purple.

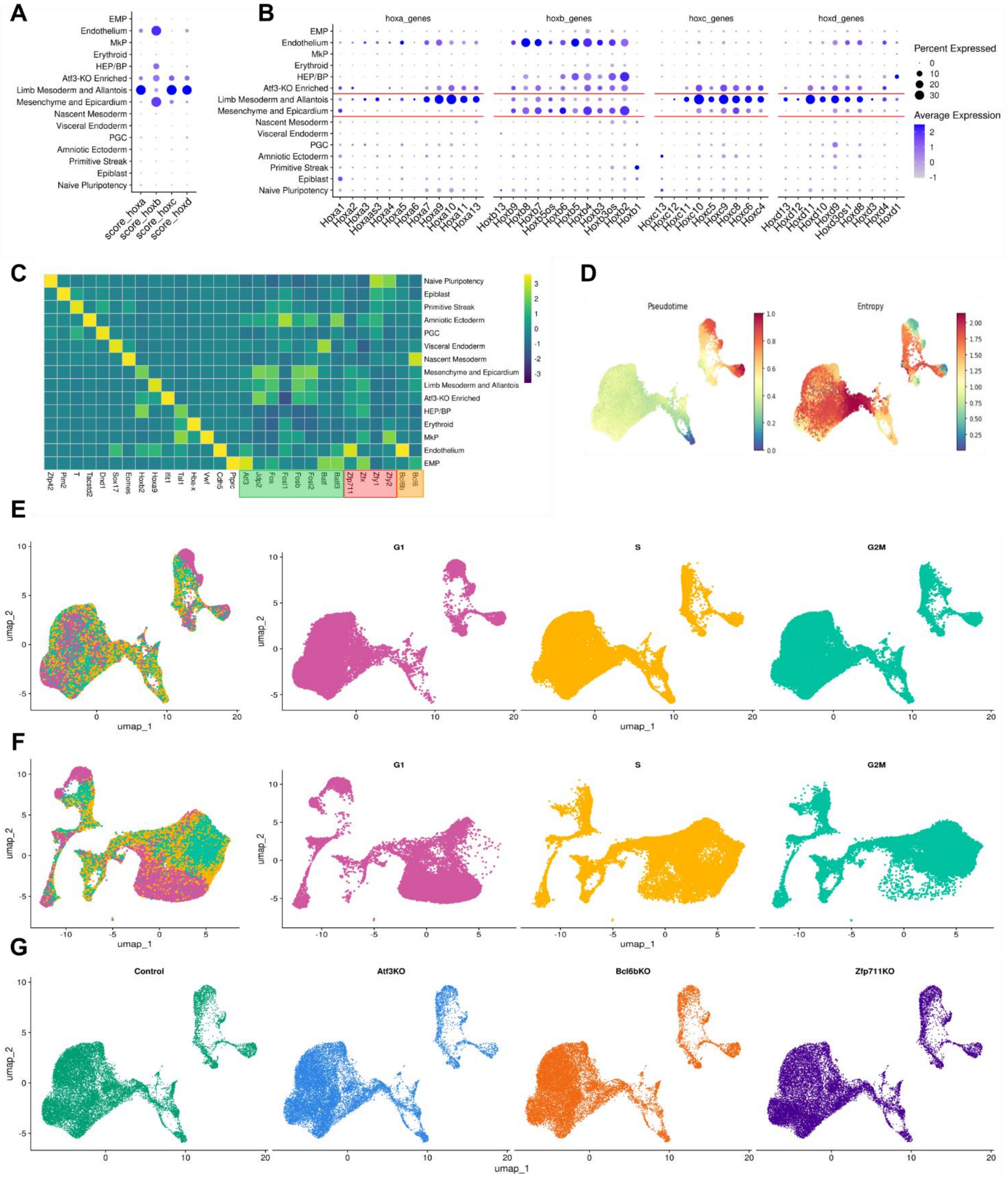

Fig. S7. Marker genes for clusters, pseudotime, and cell cycle. (A) Expression score of *Hoxa*, *Hoxb*, *Hoxc*, and *Hoxd* genes in clusters (y-axis). (B) Details of the individual *Hoxa*, *Hoxb*, *Hoxc*, and *Hoxd* genes expression in clusters (y-axis). *Hoxa*$^{+}$ Mesoderm and *Hoxb*$^{+}$ Mesoderm are highlighted with red lines. (C) The expression at the cluster level cell annotation of known marker genes, *Atf3* and some of its paralogs (green), *Zfp711* and its paralogs (red), and *Bcl6b* and its paralog (orange). D. UMAP mapping of the pseudotime from early (blue) to late (red) and entropy (differentiation potential). (E) Cell cycle phases present in cell cycle-regressed data. (F) Cell cycle phases present in non-regressed data for the cell cycle. (G) UMAPs are split by conditions.

Development • Supplementary information

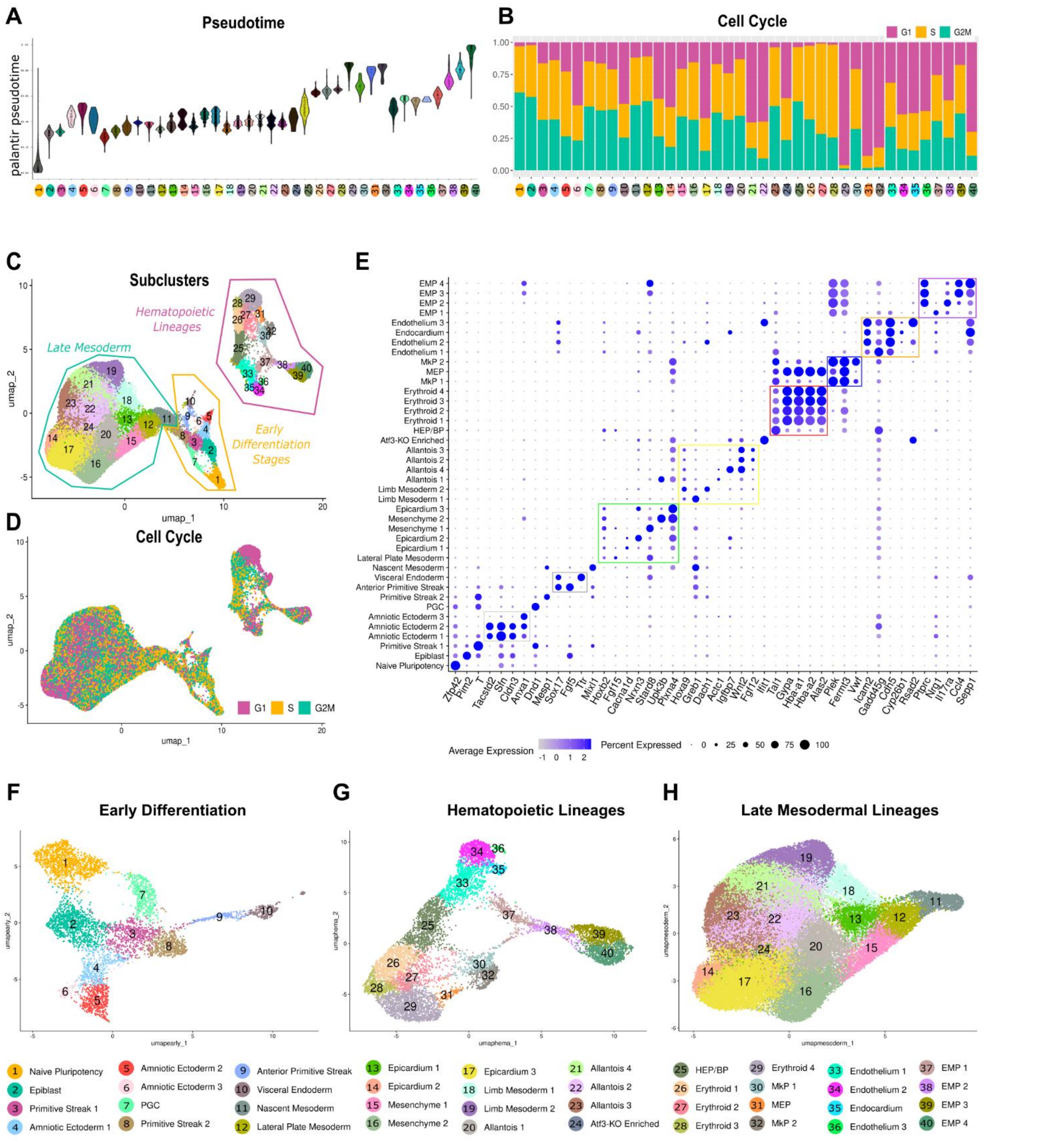

Fig. S8. Details of the data used for sub-cluster level annotation with numeric labels. (A) Pseudotime trajectory beginning from the naive sub-cluster to later stages of the differentiation. (B) Distribution of the cell cycle phases per sub-cluster. (C) UMAP shows sub-cluster level annotation (numbers) and their group-level annotation (lines). (D) Mapping of the cell cycle distribution on UMAP. (E) Marker genes are used for sub-clustering annotation (colored boxes and highlights are used as guidance to related cell types). (F-G) scRNA-seq data were divided into three subsets based on the groups, and new UMAPs were created for each.

Development • Supplementary information

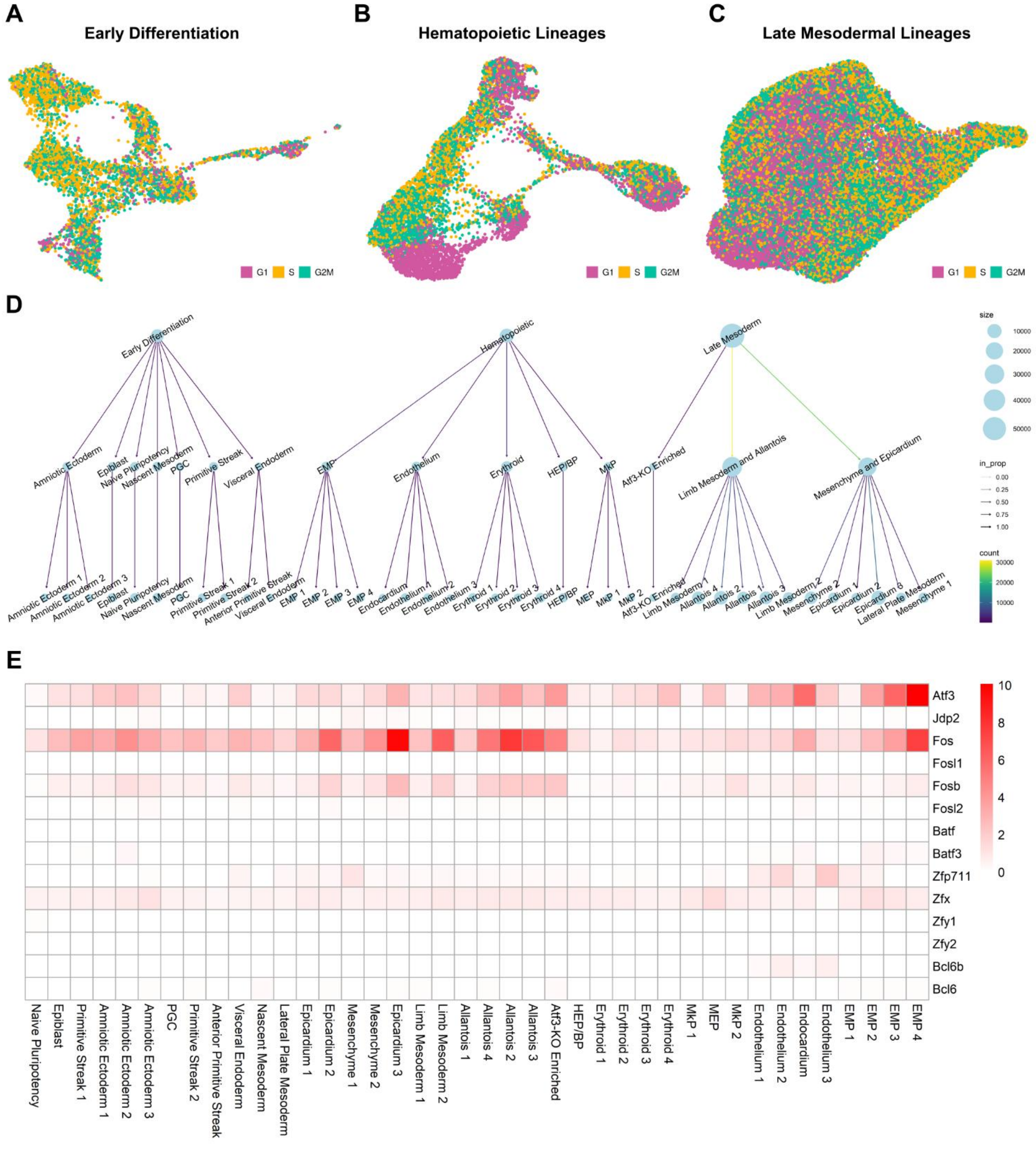

Fig. S9. Mapping the cell cycle score on group-divided UMAPs and cell type annotation hierarchy. (A-C) Cell cycle scores are mapped on group-divided UMAPs. (D) Cell type annotation hierarchy with three groups, 15 clusters, and 40 sub-clusters (the size of the circles indicates the number of cells). (E) Expression of *Atf3, Zfp711, Bcl6b,* and their paralogs at sub-cluster level.

Development • Supplementary information

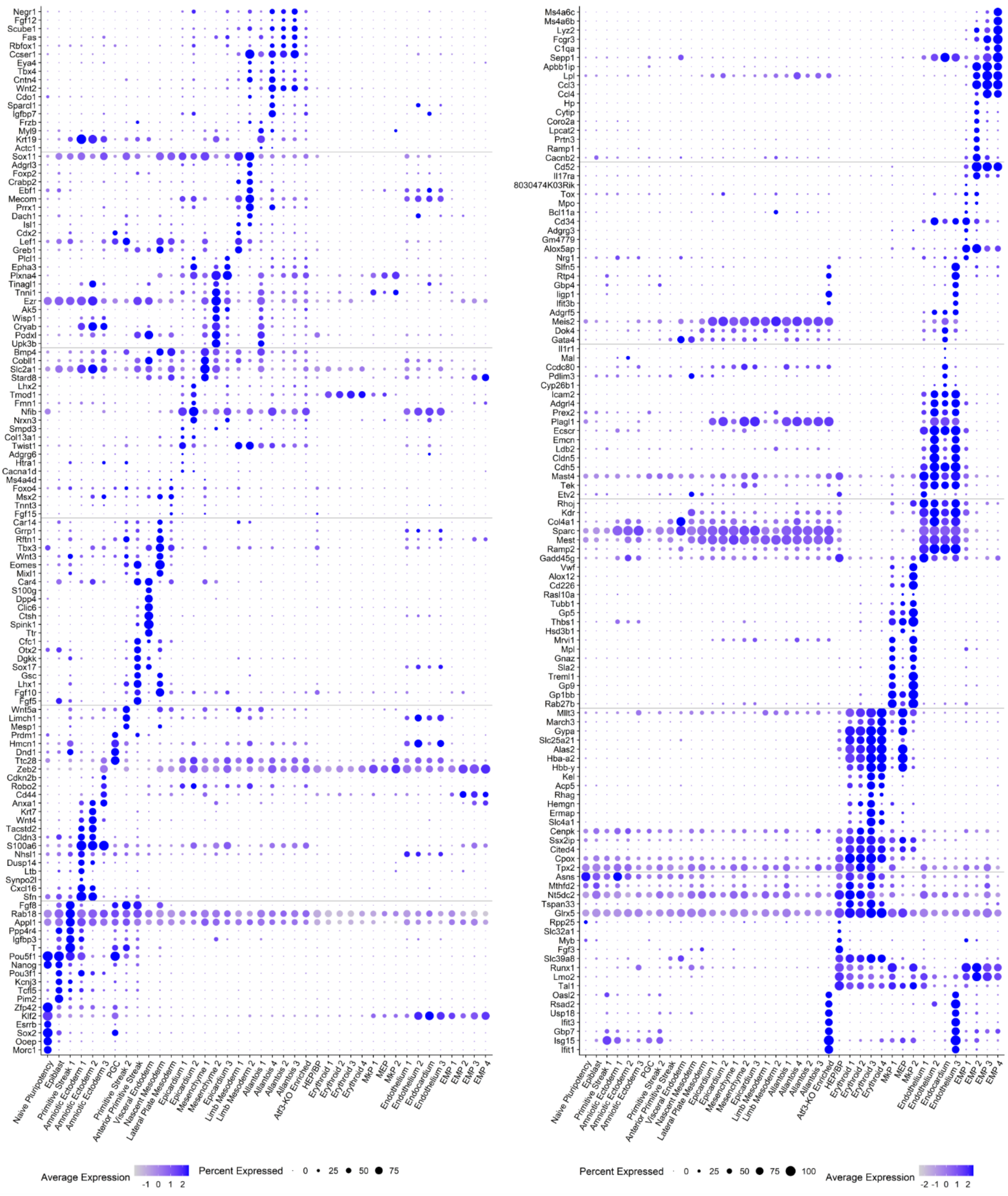

Fig. S10. Extended list of markers for sub-cluster-level annotation. Dot plots summarize expression of curated marker-gene panels across all *in vitro* subclusters.

Development • Supplementary information

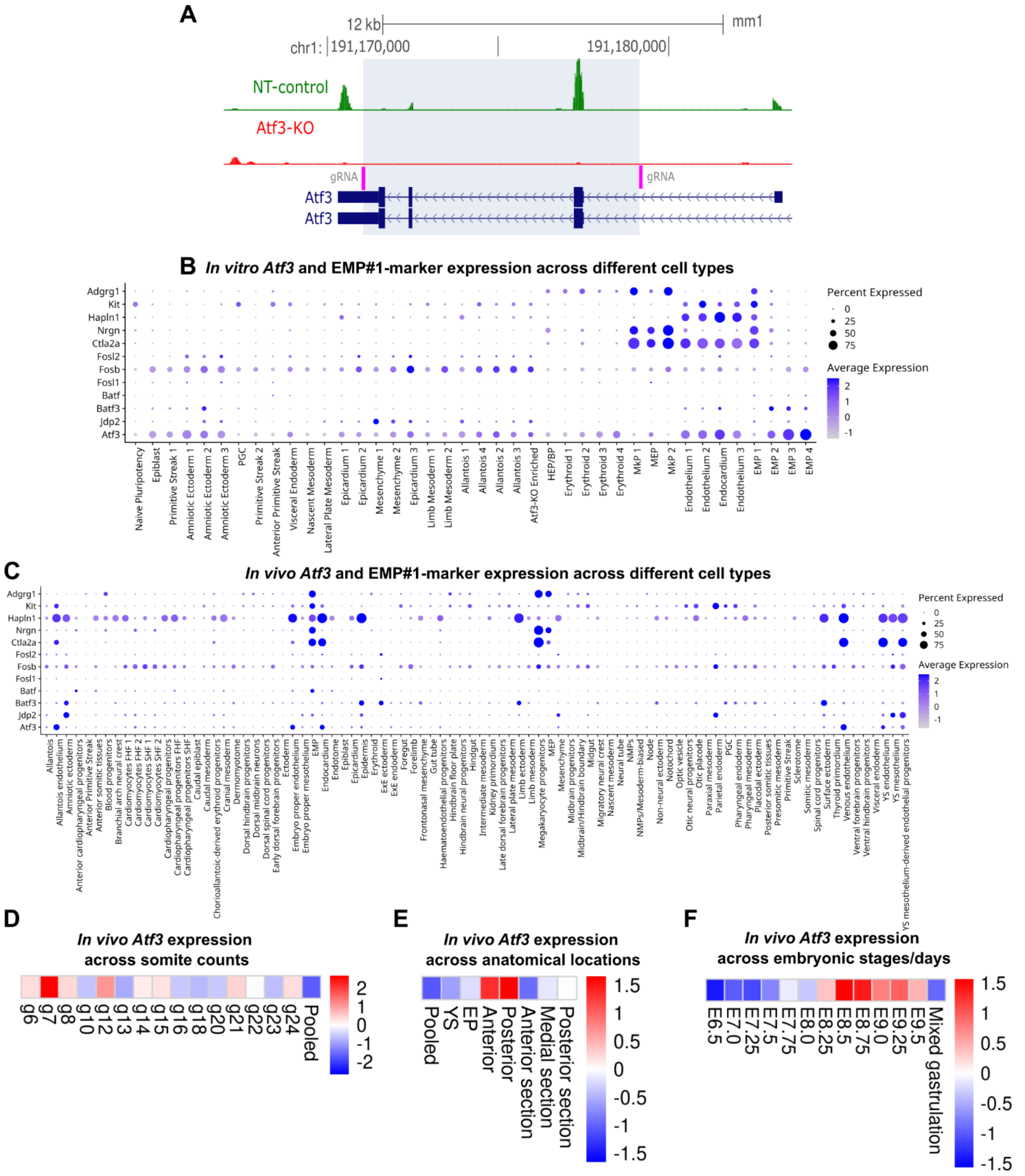

Fig. S11. *Atf3* locus targeting and expression dynamics across *in vitro* and *in vivo* datasets. (A) Genome-browser view of the *Atf3* locus. Expression reads are shown for a NT-control (green) and the *Atf3*-KO sample (red). Positions of the gRNAs used for the KO are indicated. (B) Dot plot for *Atf3*, selected AP-1/*Atf3*-paralog family members (e.g., *Jdp2, Batf3, Batf, Fos, Fosl1, Fosb, Fosl2*) and EMP#1-marker genes expression across *in vitro* subclusters. (C) The same gene set profiled for all cell types *in vivo* from the reference atlas. (D–F) *In vivo Atf3* expression summarized across (D) somite stages/counts, (E) anatomical locations, and (F) embryonic days. For all heat maps, color indicates z-scored/normalized average expression per cell type or group (red = higher, blue = lower). Abbreviations: NT, non-targeting control; KO, knockout; YS, yolk sac; EP, embryo proper.

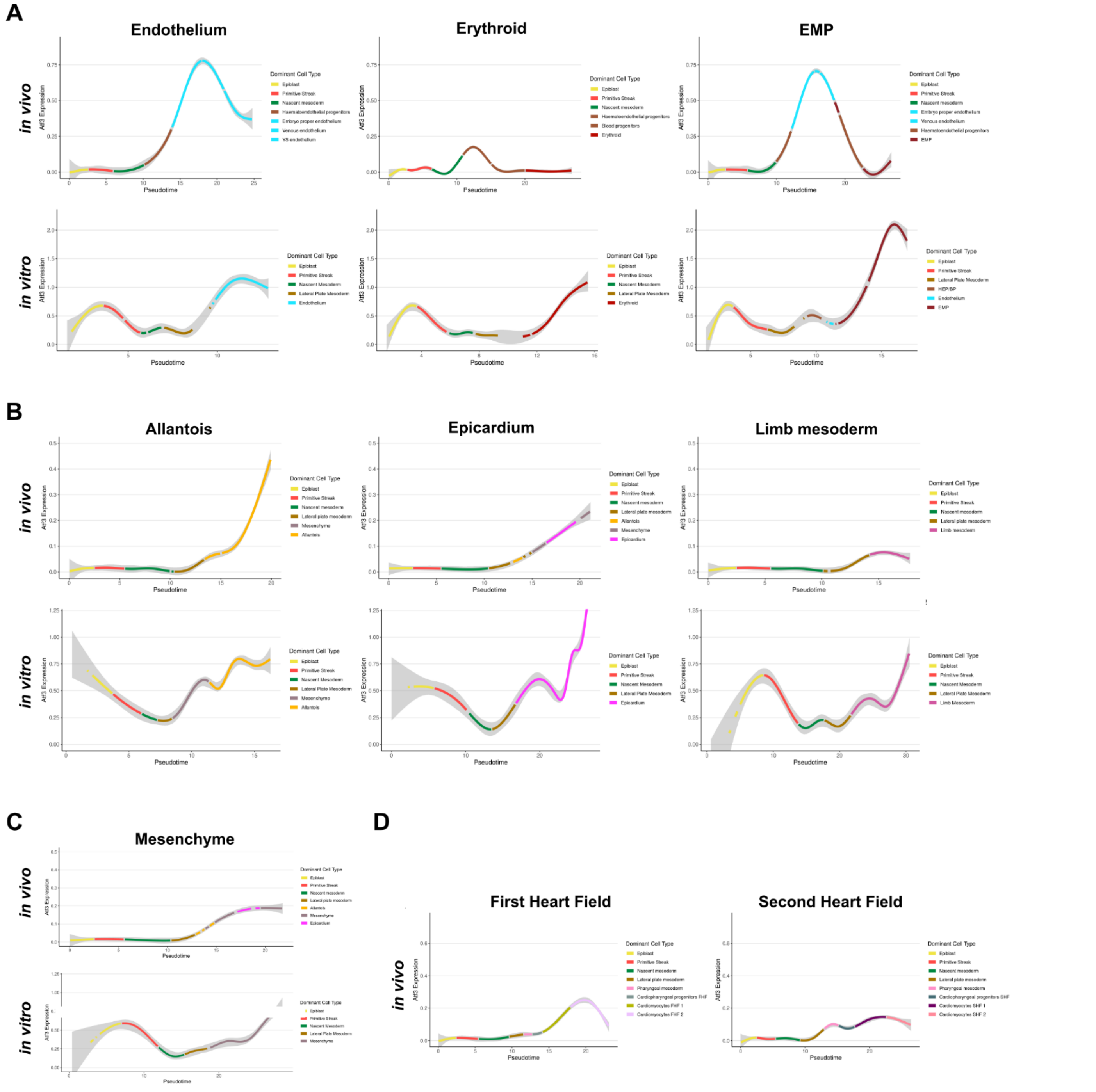

Fig. S12. ***Atf3*** **expression on developmental trajectories along pseudotime in** ***in vivo*** **and** ***in vitro*** **data.** (A–D) For each lineage, Slingshot was used to infer trajectories and compute pseudotime (x-axis). Curves show the loess-smoothed mean scaled expression (log-normalized) of lineage-specific marker sets (y-axis) along each trajectory. Shaded ribbons indicate the 95% confidence interval. Colored bars beneath each curve denote the dominant cell-type label at that pseudotime segment (legend at right). (A) Endothelium, Erythroid, and EMP trajectories shown for *in vivo* (top) and *in vitro* (bottom) datasets. (B) Allantois, Epicardium, and Limb mesoderm trajectories (*in vivo* top; *in vitro* bottom) showing matched rises of lineage-specific programs across systems. (C) Mesenchyme trajectory (*in vivo* top; *in vitro* bottom). (D) First and Second Heart Field trajectories (*in vivo* only).

Development • Supplementary information

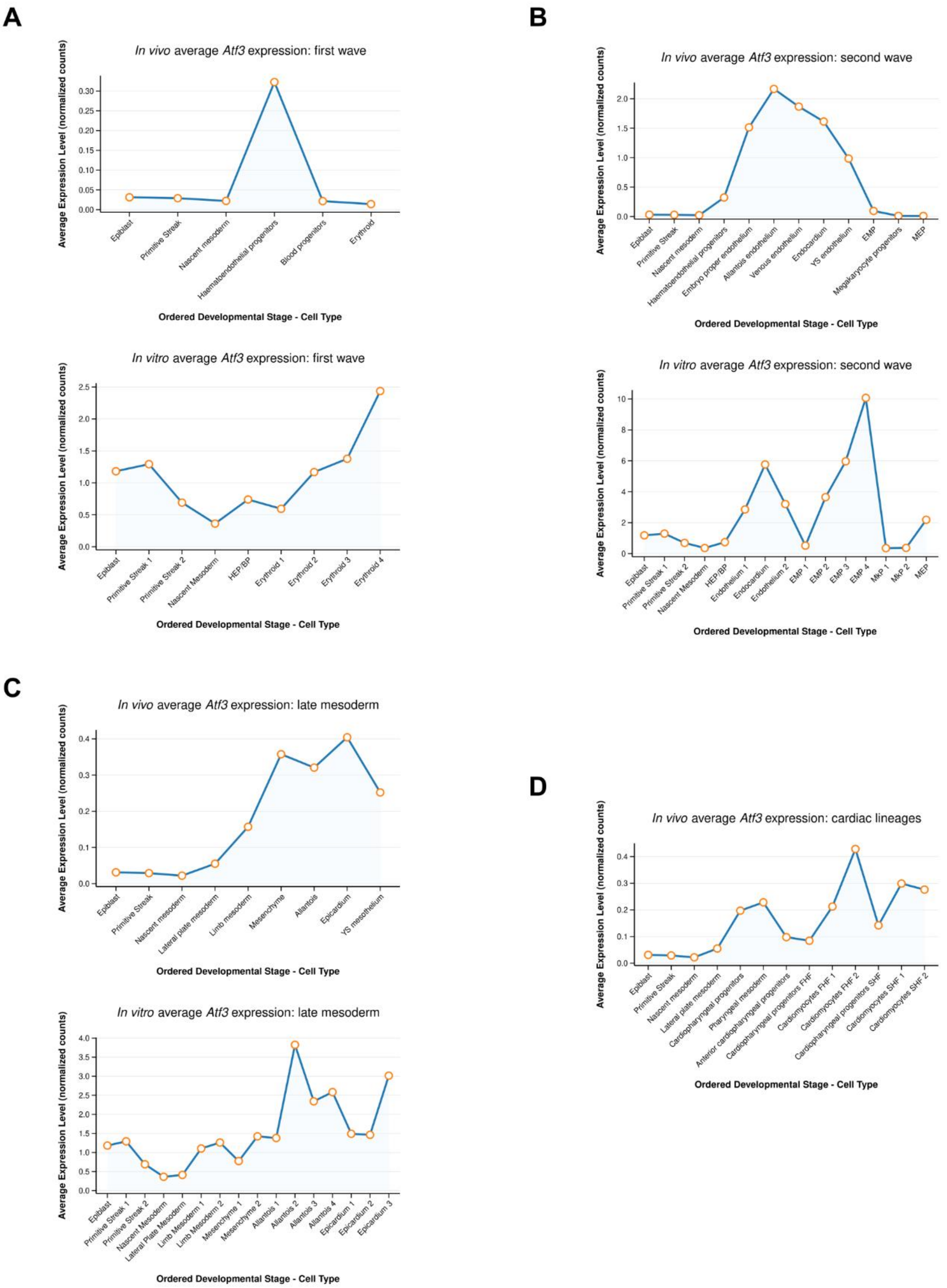

Fig. S13. Average *Atf3* expression on developmental trajectories along pseudotime in *in vivo* and *in vitro* data (after manual ordering of cell types). (A) Hematopoietic primitive wave trajectory shown for *in vivo* (top) and *in vitro* (bottom) datasets. (B) Hematopoietic second wave trajectory (*in vivo* top; *in vitro* bottom). (C) Late mesoderm trajectory (*in vivo* top; *in vitro* bottom). (D) Cardiac trajectory (*in vivo* only).

Development • Supplementary information

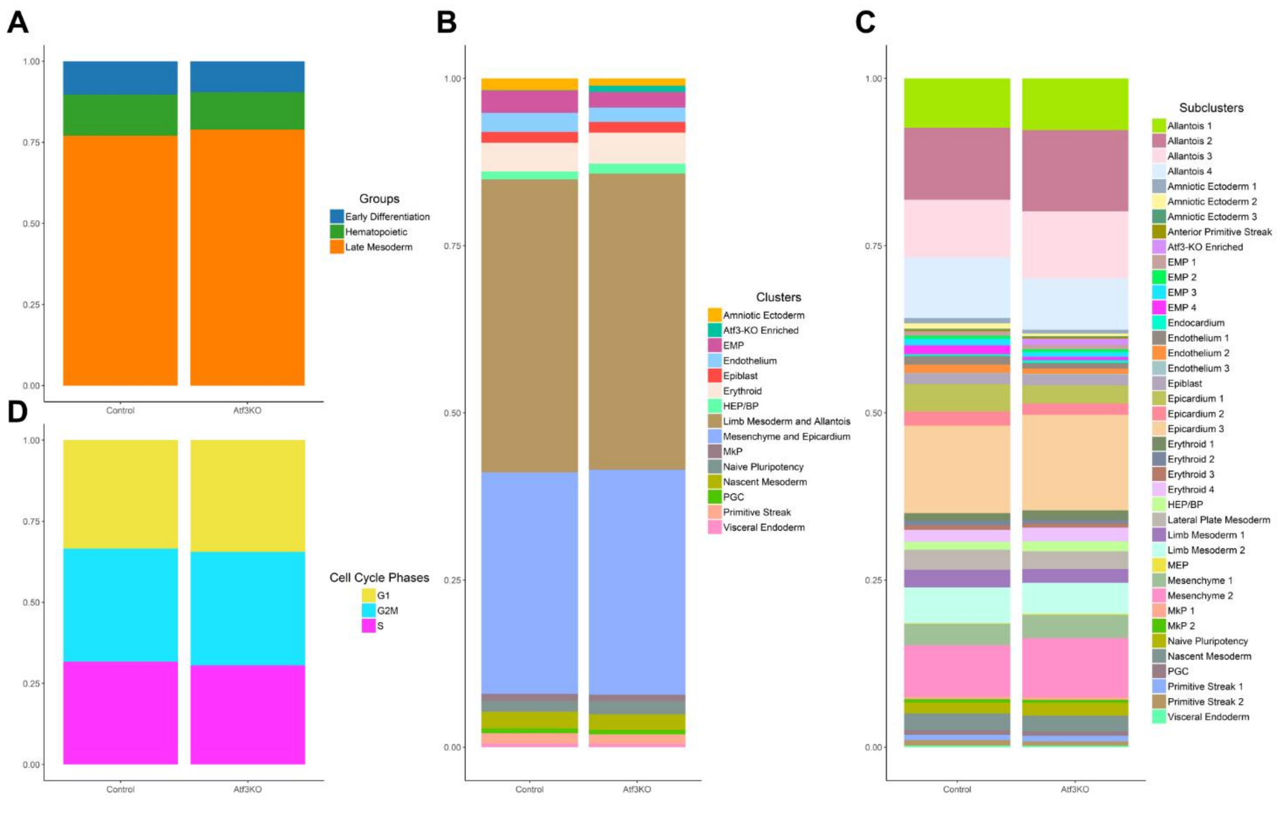

Fig. S14. Δ*Atf3* cells: Differential Abundance Analysis results using Speckle R Package. (A-D) Fraction of annotated cell types in groups (A), clusters (B), sub-clusters (C), and cell cycle (D).

Development • Supplementary information

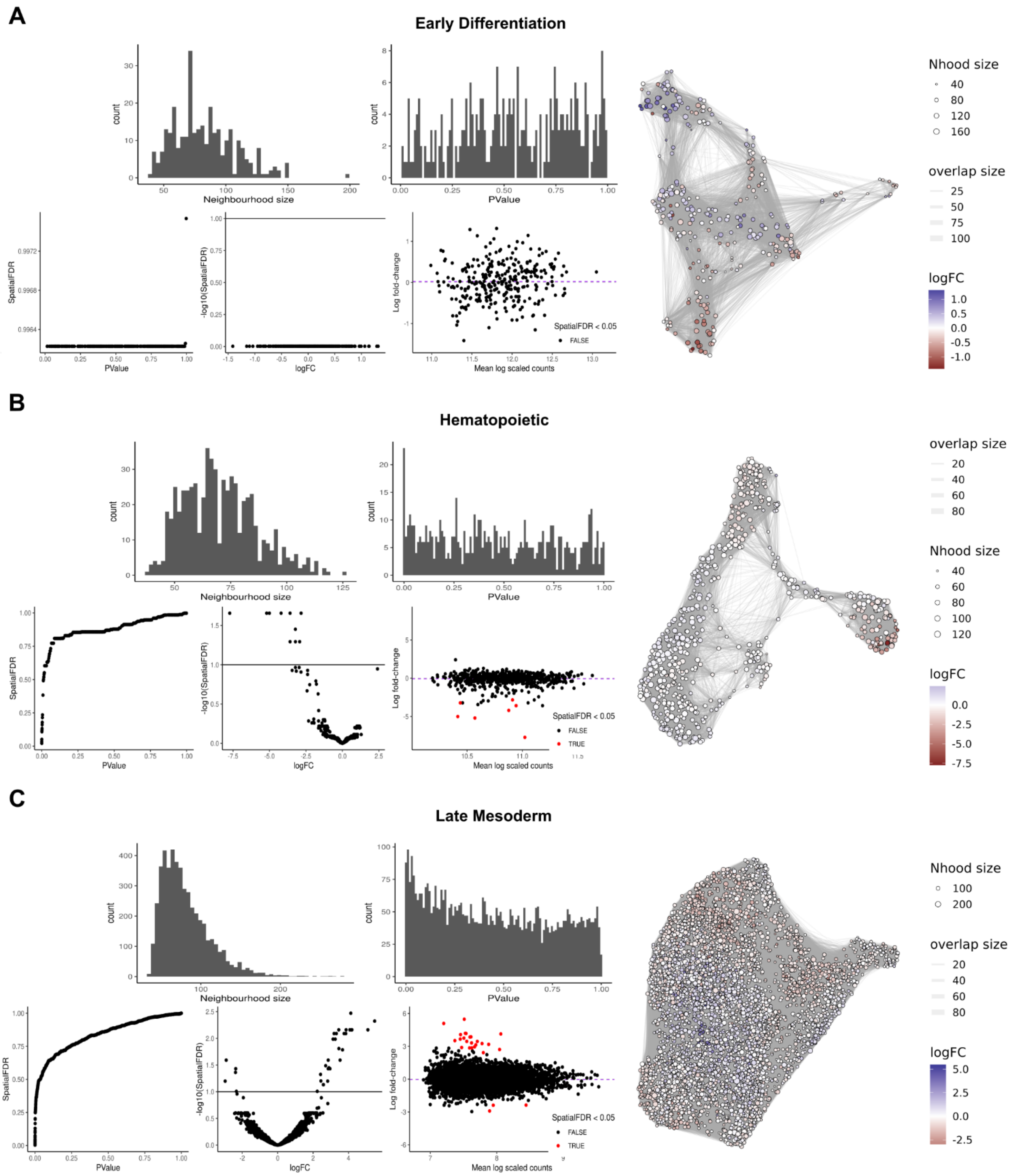

Fig. S15. Quality control and visualization of MiloR differential abundance analysis for *Atf3*-KO. (A) Early Differentiation group, (B) Hematopoietic group, and (C) Late Mesoderm group. For each comparison, the plots show: the neighborhood graph colored by log fold change (logFC), with node size scaled by cell count; histograms of neighborhood sizes and p-values; the relationship between p-values and the spatially-corrected FDR (SpatialFDR); a volcano plot (logFC vs. −log10,SpatialFDR); and an MA plot (logFC vs. mean abundance). In relevant plots, significant neighborhoods (SpatialFDR < 0.05) are highlighted in red. UMAP with neighborhood overlay (node size = neighborhood size. Color scale indicates log fold-change (red = decreased, blue = increased in Δ*Atf3*).

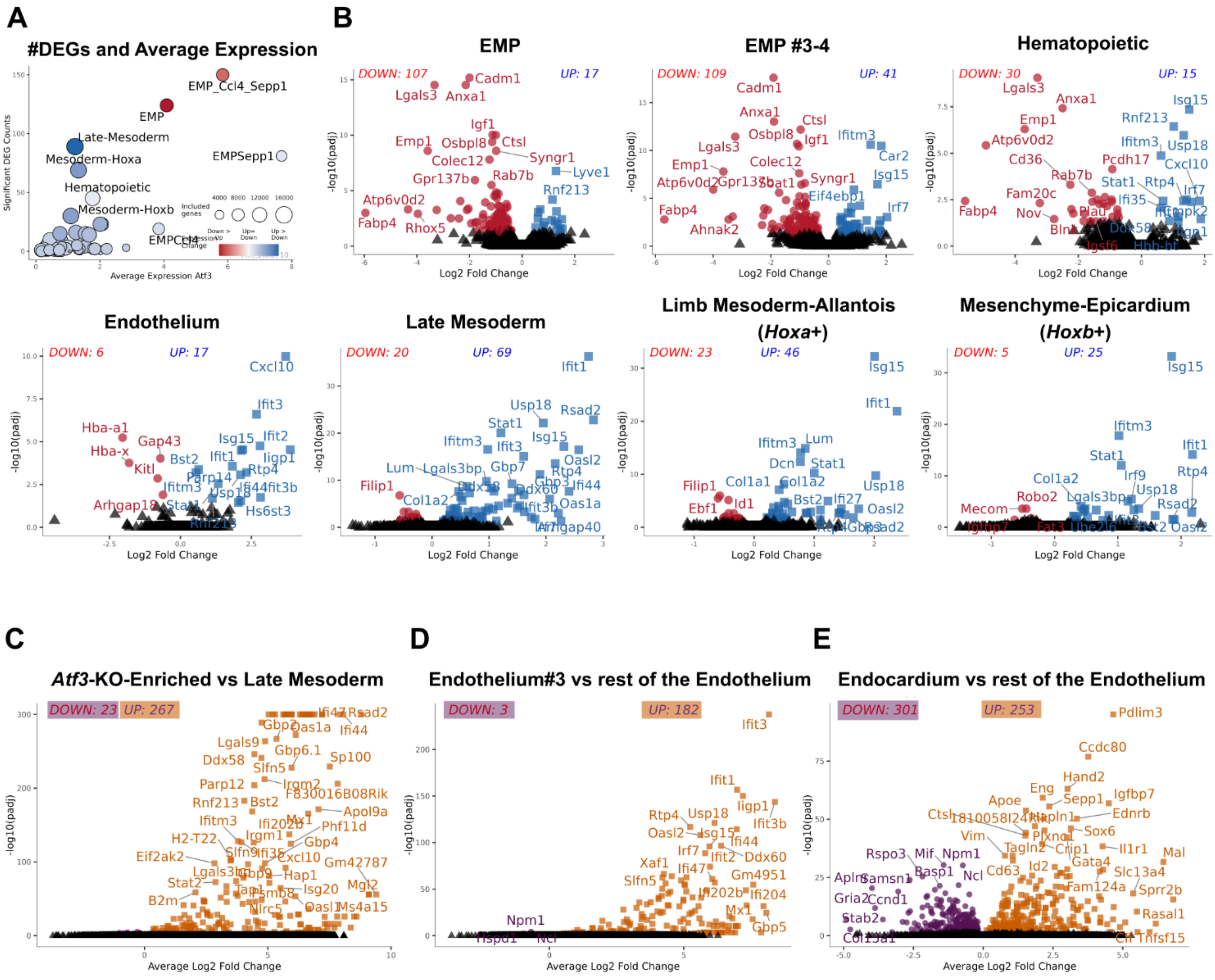

F

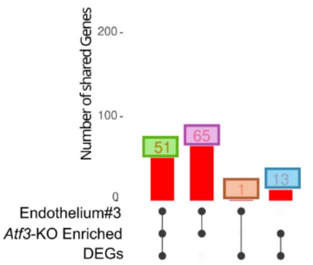

***Atf3*-KO-Enriched, Endothelium#3 and Endocardoum intersection**

Bst2	Eif2ak2	Gm4951	Ifih1	Ifitm3	Irgm2	Mitd1	Parp14	Sp100	Tor3a
Cmpk2	Gbp2	H2-D1	Ifit1	Igtp	Isg15	Oas1a	Rnf213	Stat1	Trim25
Cxcl10	Gbp2b	H2-T22	Ifit2	Iigp1	Lgals3bp	Oasl1	Rsad2	Stat2	Trim30a
Ddx58	Gbp3	Ifi35	Ifit3	Irf7	Lgals9	Oasl2	Rtp4	Tmem106a	Ube2l6
Ddx60	Gbp7	Ifi44	Ifit3b	Irgm1	Ly6e	Parp12	Samd9l	Tmsb10	Usp18
Xaf1	Hmga2	Irf9	Adar	B2m	Dtx3l	Gm12185	Hap1	Ifi204	Ly6a
Apobec1	Id1	Lgals1	Apol10b	Cd274	Gbp4	Gm44148	Helz2	Ifi47	Ly6c1
Atf3	Ifi27	Lum	Apol9b	Daxx	Gbp5	Gm4841	Herc6	Isg20	Mx1
Cd63	Ifi27l2a	Sparc	Ascc3	Ddx24	Gbp8	H2-K1	Hspd1	Itm2b	Ncl
Emp3	Igfbp7	Lyve1	Atp10a	Dhx58	Gbp9	H2-Q4	Ifi202b	Lgals8	Nlgn2
Nmi	Parp10	Prdx5	Psmb9	Psme2b	Shisa5	Tap1	Tdrd7	Trim21	Zbp1
Npm1	Parp9	Psmb10	Psme1	S100a13	Slfn2	Tap2	Tmem140	Trim34a	Zc3hav1
Olfr56	Phf11b	Psmb8	Psme2	Sepw1	Slfn5	Tapbp	Tnfsf10	Vmn2r94	9930111J21Rik2

Fig. S16. Δ*Atf3* cells: Differential Gene Expression Analysis and Unique Cluster Markers. (A) shows the average expression level and number of the significant DEGs (adjusted-P < 0.05) in groups, clusters, and sub-clusters. The size of the circles indicates the number of genes included in the analysis after removing low-expressed genes in the group, cluster, or sub-cluster. The circle color indicates up- or downregulation (blue and red respectively). (B) shows the up- or downregulation of genes in multiple conditions as a volcano plot. (C-E) Marker genes of the small/unique sub-clusters are Δ*Atf3*-Enriched, Endothelium#3, and Endocardium. (F) The number of shared genes between Endothelium#3, Δ*Atf3*-Enriched, and DEGs with color code. Supporting data are shown in Table S4A-K.

Development • Supplementary information

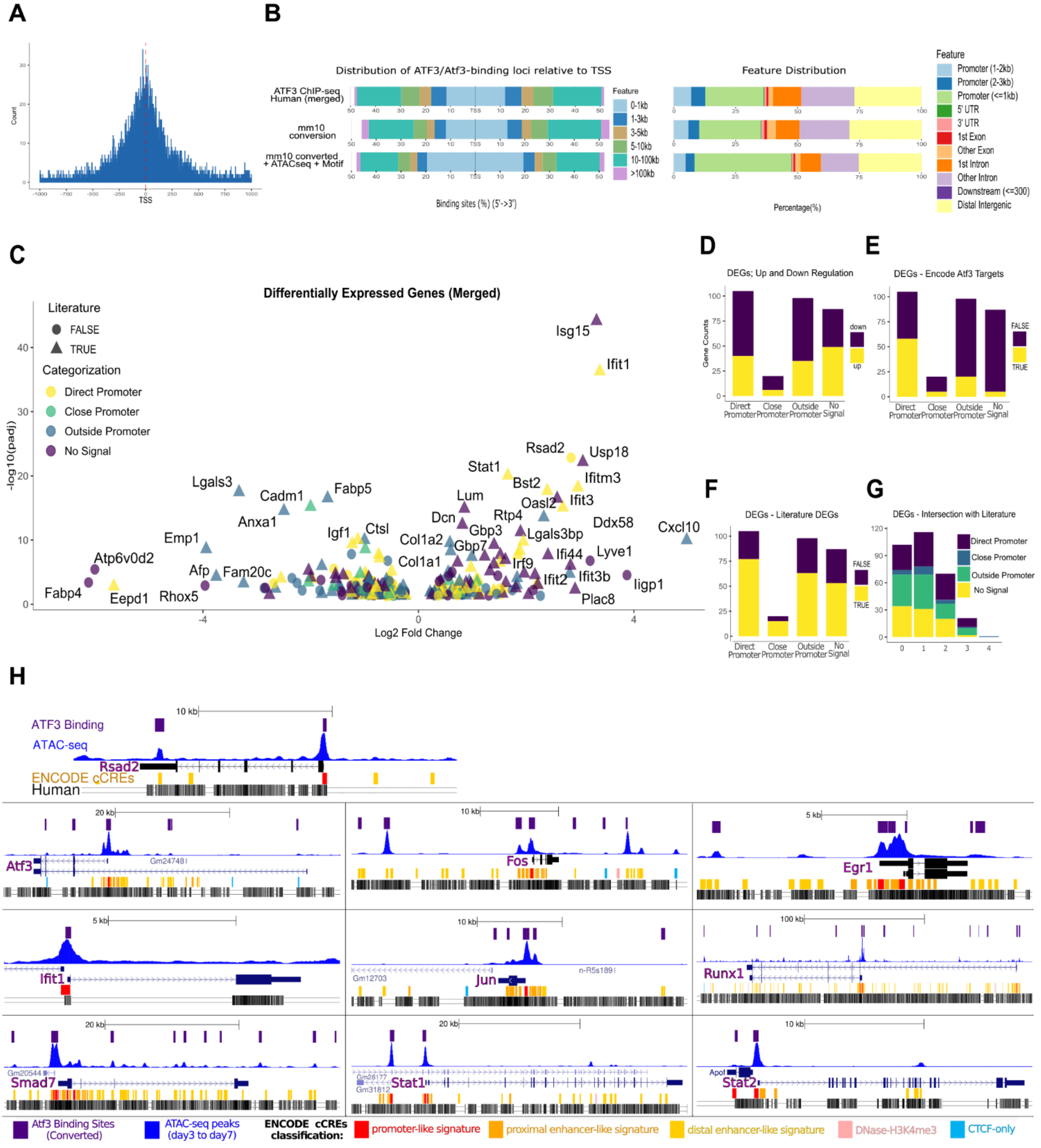

Fig. S17. Results and categorization of the Differential Gene Expression Analysis of the Δ*Atf3* cells. (A) *Atf3* binding regions distribution histogram relative to the Transcription Start Site (TSS). (B) *Atf3* ChIP-seq peak regions location and distance to TSS in three stages of the analysis going from human to mouse. (C) Volcano plot of all the significant DEGs. (D-G) Distribution of the DEGs, *Atf3*-binding categorization, TF Targets dataset, and DEGs found in the literature. (H) Some examples of *Atf3* binding sites with ATAC-seq peaks in three example DEGs. Supporting data can be seen in Table S4A-K, S6A, and S7A-E.

Development • Supplementary information

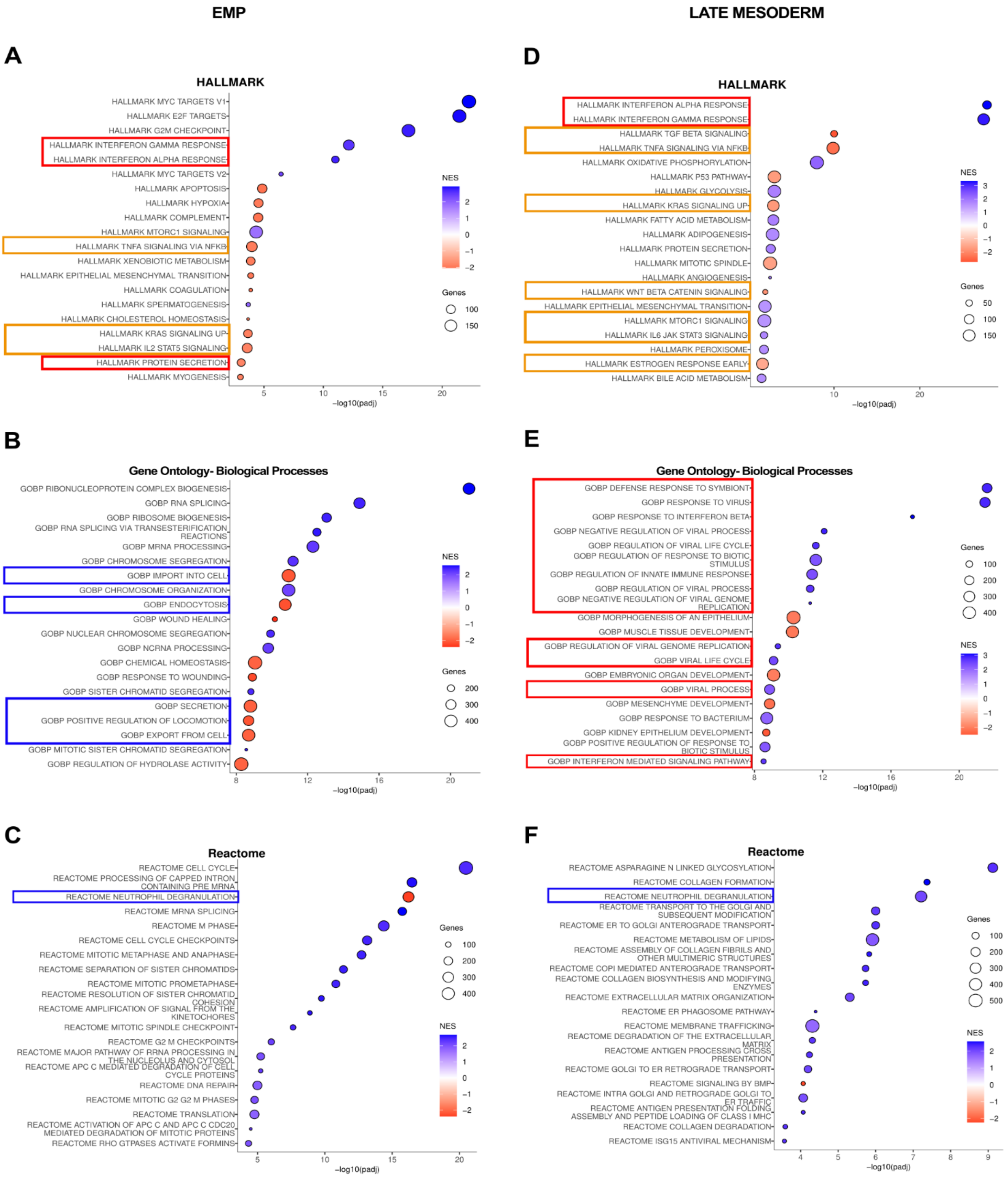

Fig. S18. GSEA results of the differentiated Δ*Atf3* cells on EMP and Late Mesoderm. (A-C) Show the significance (-log10 (adjusted-P)) of the GSEA for three collections of gene sets (from MSigDB [https://www.gsea-msigdb.org/]) in EMP and the top 20 gene sets in that collection (on the left). The size of the circle indicates the number of genes in a gene set (after removal of the genes that are expressed at a very low level). Blue to red indicates the increase or decrease of the Normalized Enrichment Score. Red-colored boxes highlight "interferon and antiviral related pathways," blue-colored boxes highlight "degranulation, cellular transport, and secretion related pathways," and orange-colored boxes highlight "signaling pathways." (D-F) Show GSEA results similar to panels A-C for Late Mesoderm.

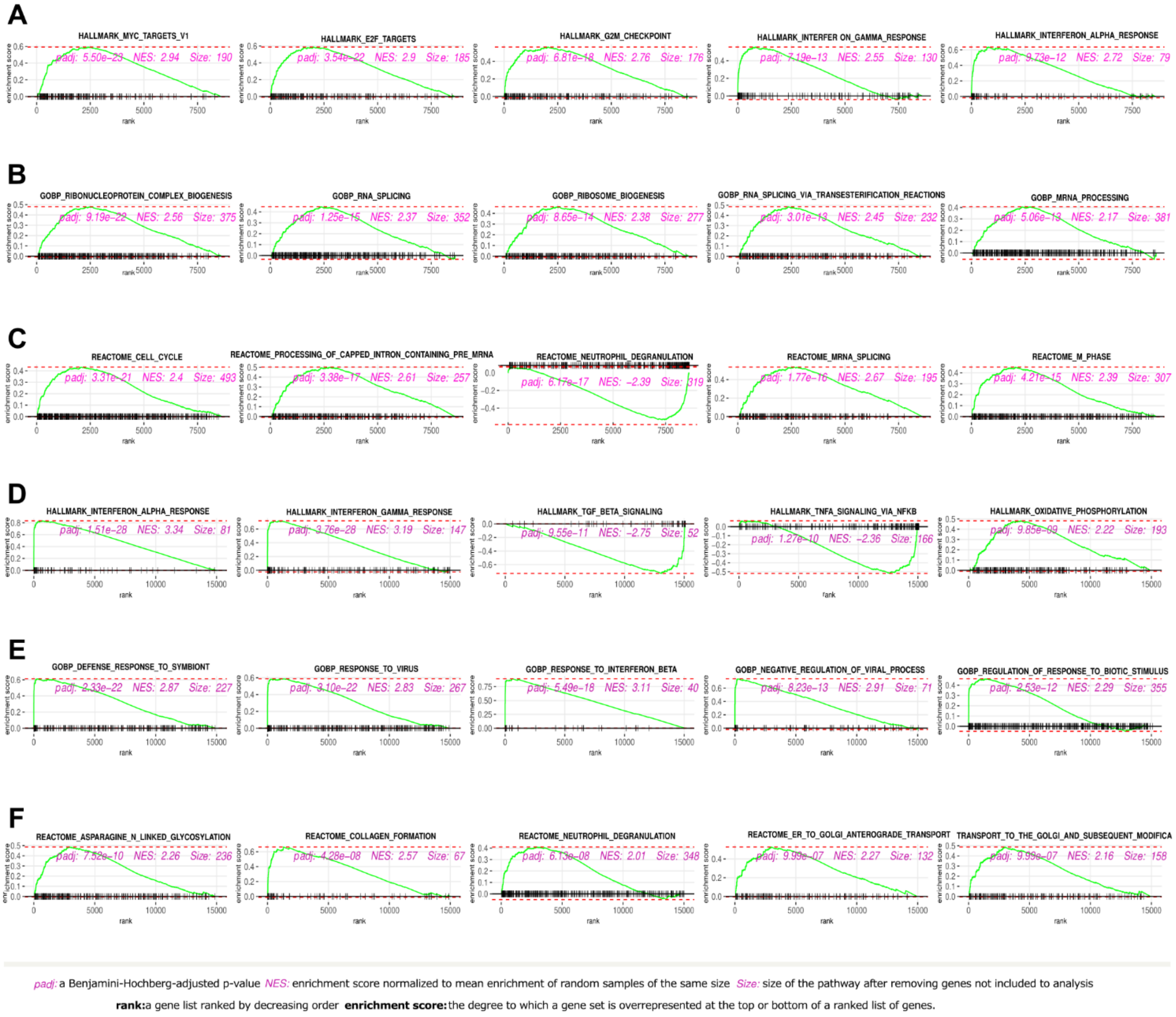

Fig. S19. GSEA Results of the Δ*Atf3* cells on EMPs and Late Mesoderm. (A-C) show enrichment plots of the top-5 results on EMPs. (D-F) show for Late Mesoderm the GSEA results, similar to panels A-C. Supporting data can be seen in Table S5A-F.

Development • Supplementary information

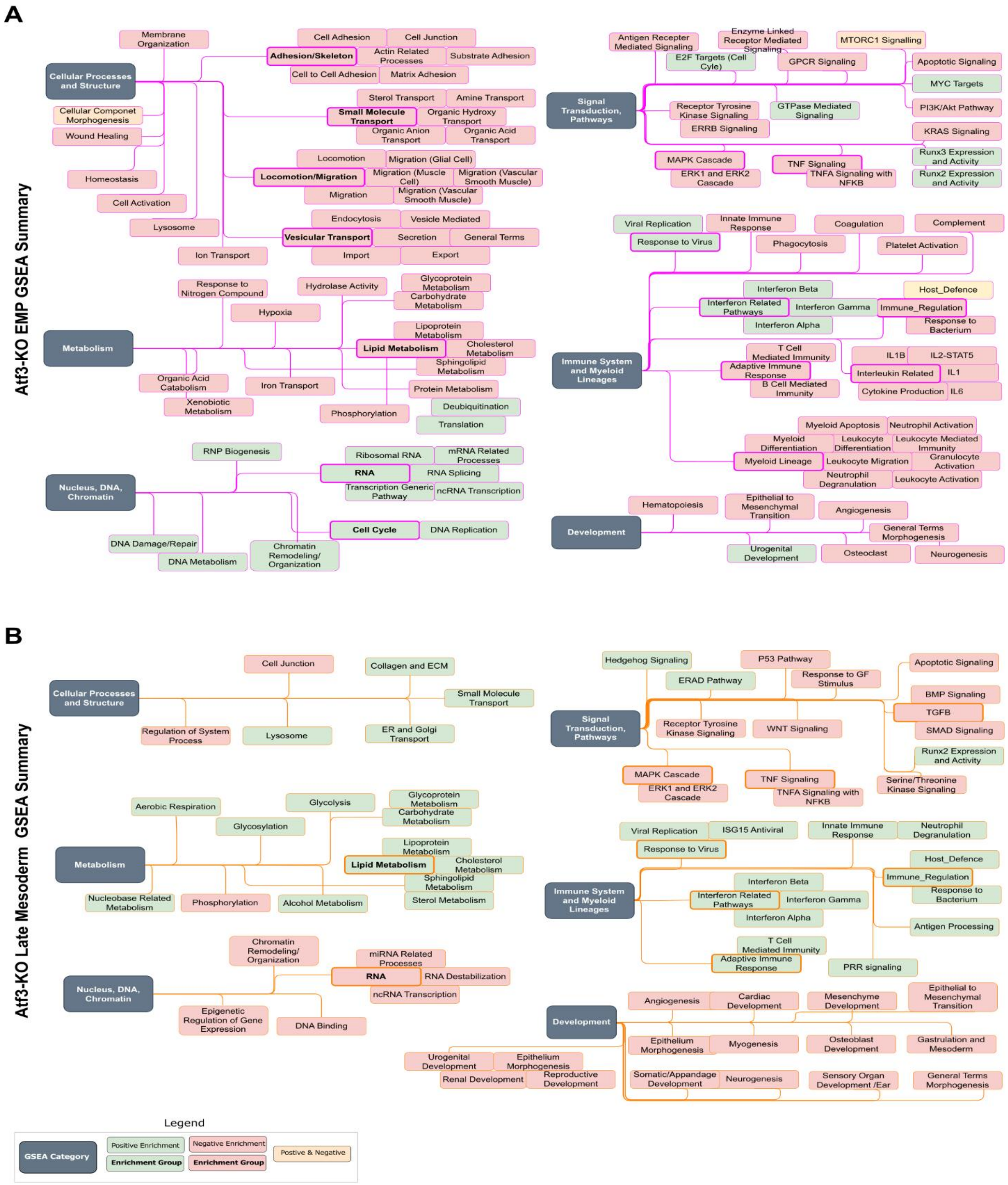

Fig. S20. Manually grouped GSEA results of the Δ*Atf3* cells. (A) shows the categorization GSEA results of EMPs. (B) the categorization of Late Mesoderm GSEA results. Supporting data can be seen in Table S5A-G.

Development • Supplementary information

A

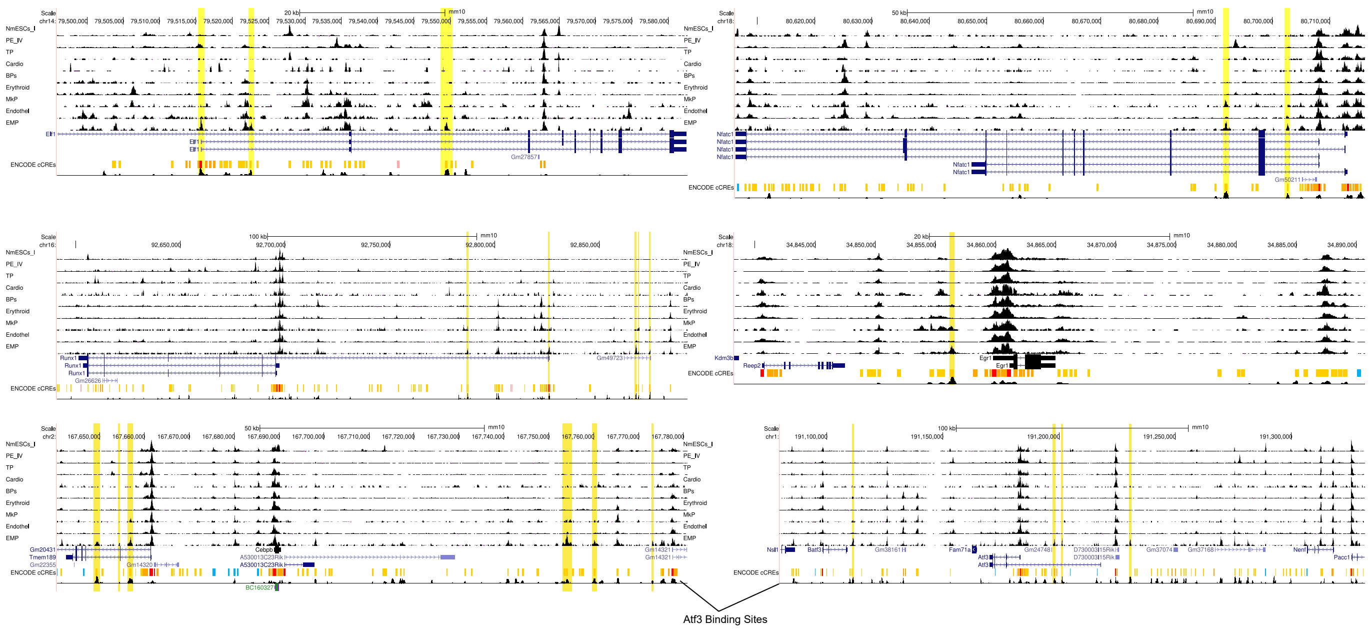

B

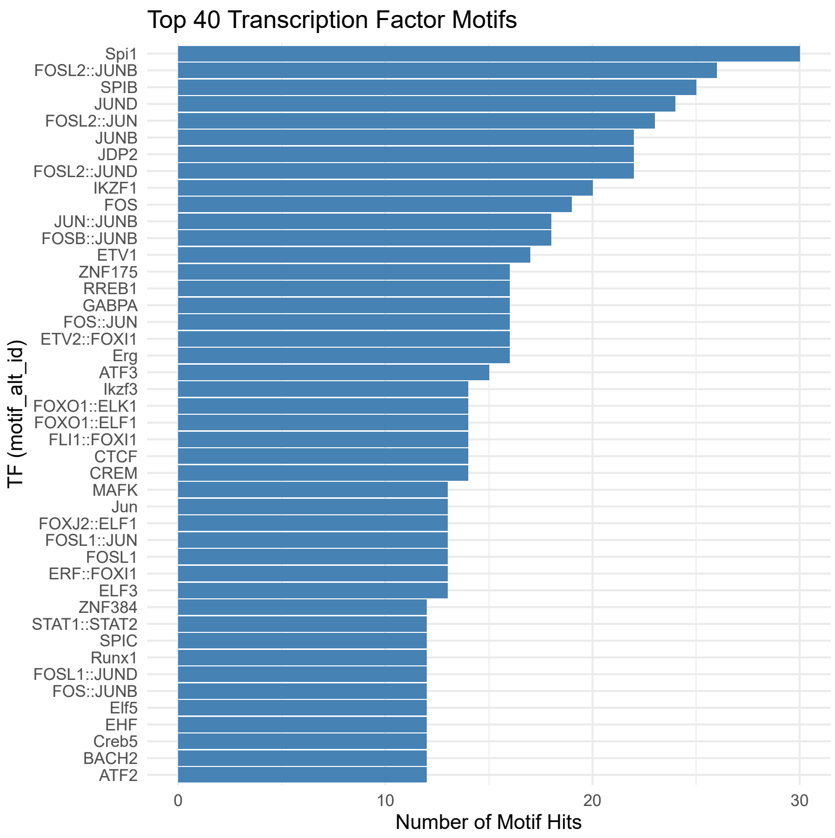

C

```
chr1 191197998 191198148
chr1 191201349 191201448
chr1 191230681 191230838
chr1 191111382 191111512
chr18 34856350 34856457
chr12 85468806 85468900
chr12 85385795 85385932
chr12 85381807 85381940
chr7 19338181 19338277
chr7 141268993 141269105
chr16 92825964 92826068
chr16 92867431 92867525
chr16 92867327 92867554
chr16 92874170 92874295
chr16 92868669 92868813
chr16 92787160 92787319
chr2 45049476 45049565
chr10 120541727 120541879
chr10 120616681 120616839
chr12 25213955 25214126
chr15 50867025 50867148
chr13 83609303 83609486
chr12 85710751 85710877
chr19 37436607 37436748
chr19 37503095 37503289
chr19 37495346 37495497
chr2 167649364 167649523
chr2 167656669 167656822
chr2 167654413 167654549
chr2 167753957 167754109
chr2 167760105 167760247
chr2 167773026 167773149
chr14 79549602 79549735
chr14 79515620 79515714
chr14 79522495 79522609
chr9 32500981 32501132
chr11 11667659 11667766
chr6 134115716 134115853
chr8 120720001 120720131
chr8 120686234 120686375
chr8 120716151 120716264
chr1 52022090 52022349
chr18 80691862 80692020
chr18 80738323 80738440
chr18 80739053 80739158
chr18 80702581 80702698
chr6 16898532 16898723
chr6 16891970 16892139
```

Fig. S21. Newly accessible chromatin regions in delayed EMP maturation in *Atf3*-KO. (A) Emergence of new chromatin-accessible regions in EMP Cells. This panel shows six selected genes important for EMP development, which also exhibit ATF3 binding according to publicly available data. (B) Motif analysis of these peaks reveals that *Spi1* is the most common transcription factor with a motif present in these regions. (C) The genomic coordinates of the identified peaks. The data were produced from single-cell RNA sequencing (scRNA-seq) and single-cell ATAC sequencing (scATAC-seq) of *in vitro* hemato-endothelial differentiation. The coordinates of the peaks for Naive mESCs (NmESCs_I), parietal endoderm (PE_IV), thyroid primordium (TP), and blood progenitors (BPs) were obtained from a separate study. (Manuscript in preparation).

Development • Supplementary information

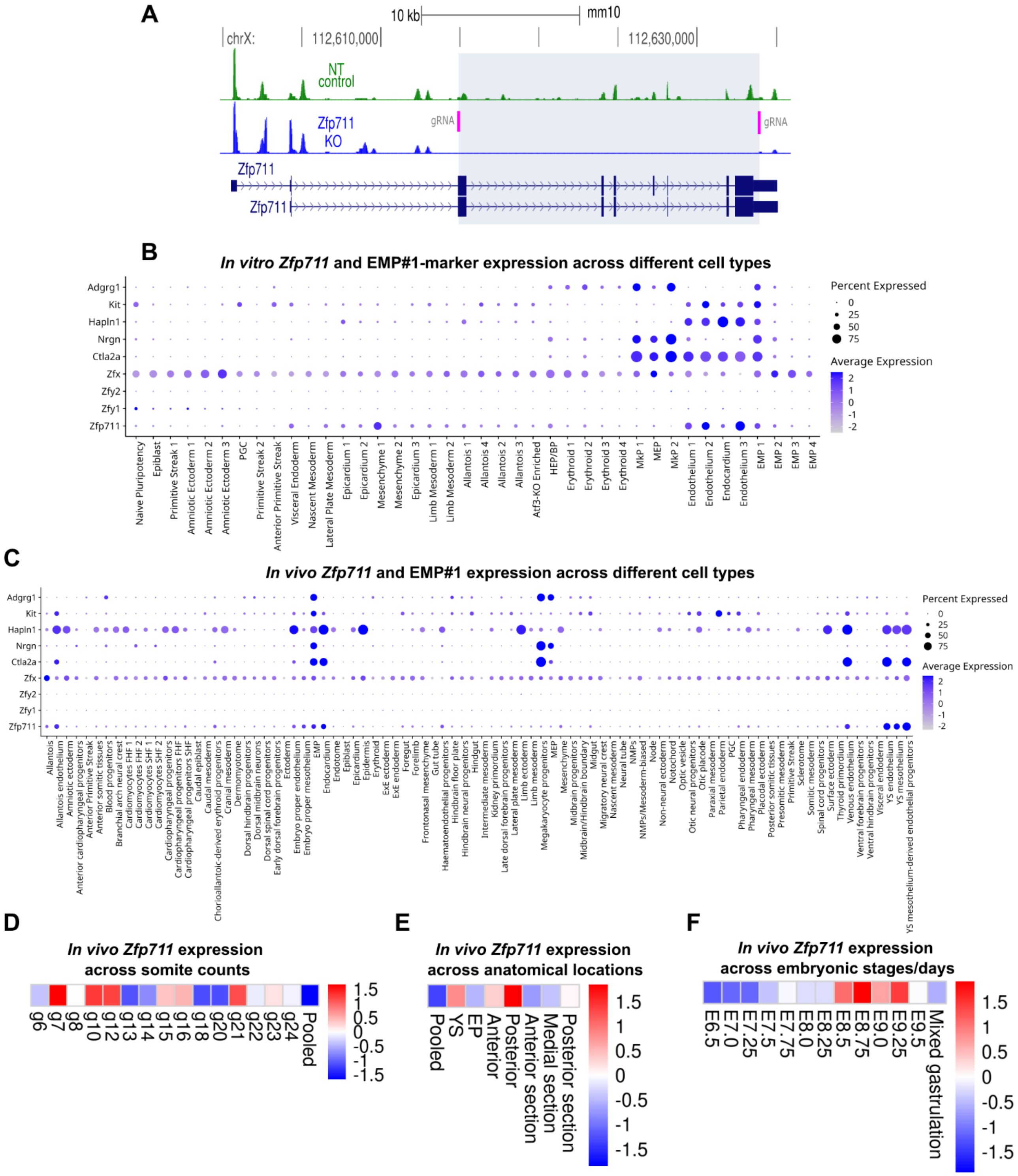

Fig. S22. *Zfp711* locus targeting and expression dynamics across *in vitro* and *in vivo* datasets. (A) Genome-browser view of the *Zfp711* locus. Expression reads are shown for a NT-control (green) and the *Zfp711*-KO sample (red). Positions of the gRNAs used for the KO are indicated. (B) Dot plot of expression for *Zfp711*, *Zfp711*-paralogs and EMP#1-marker genes across *in vitro* subclusters. (C) The same gene set profiled for all cell types *in vivo* from the reference atlas. (D–F) *In vivo Zfp711* expression summarized across (D) somite stages/counts, (E) anatomical locations, and (F) embryonic days. For all heat maps, color indicates z-scored/normalized average expression per cell type or group (red = higher, blue = lower). Abbreviations: NT, non-targeting control; KO, knockout; YS, yolk sac; EP, embryo proper.

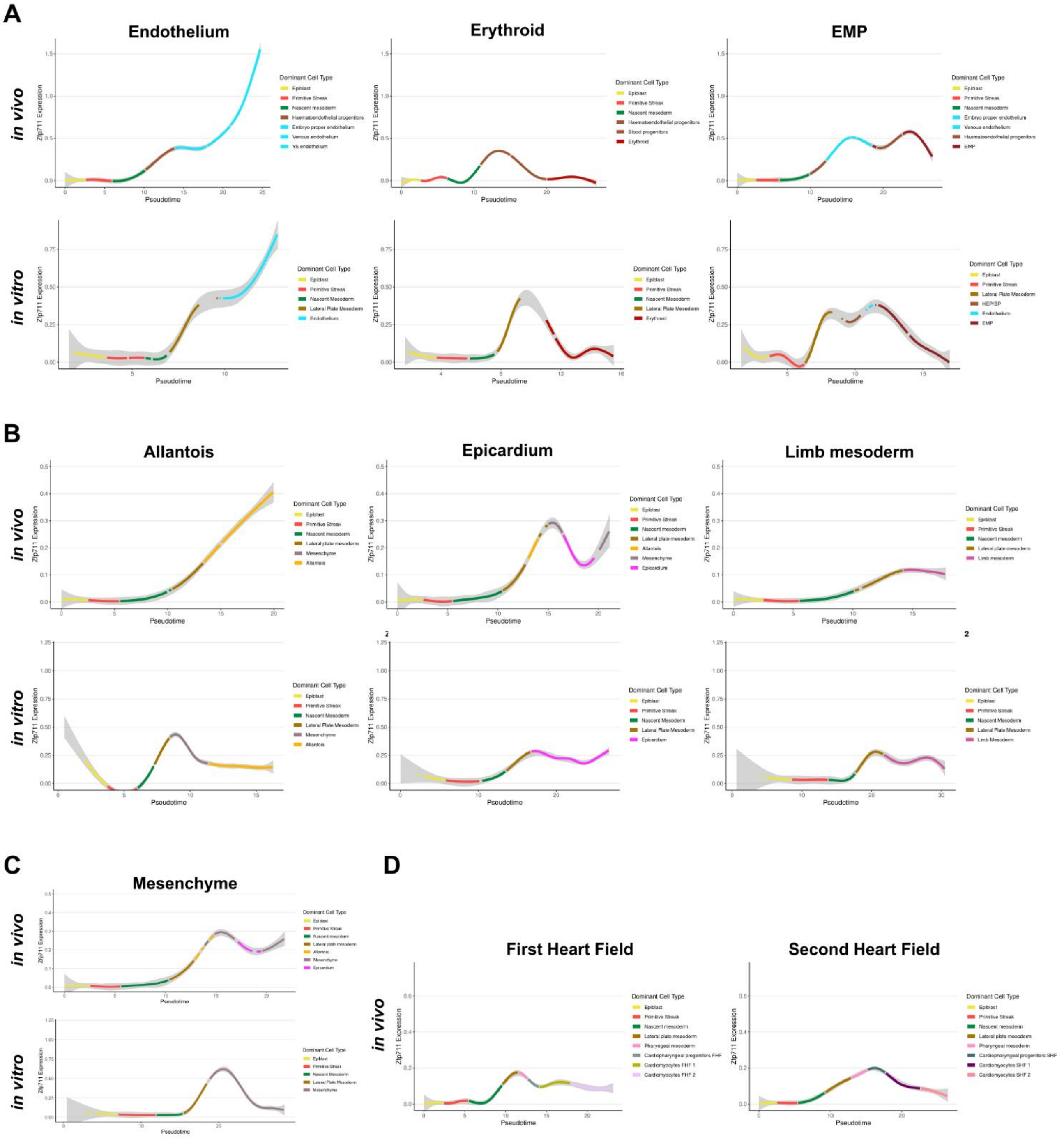

Fig. S23. ***Zfp711*** **expression on developmental trajectories along pseudotime in** ***in vivo*** **and** ***in vitro*** **data .** (A–D) For each lineage, Slingshot was used to infer trajectories and compute pseudotime (x-axis). Curves show the loess-smoothed mean scaled expression (log-normalized) of lineage-specific marker sets (y-axis) along each trajectory. Shaded ribbons indicate the 95% confidence interval. Colored bars beneath each curve denote the dominant cell-type label at that pseudotime segment (legend at right). (A) Endothelium, Erythroid, and EMP trajectories shown for *in vivo* (top) and *in vitro* (bottom) datasets. (B) Allantois, Epicardium, and Limb mesoderm trajectories (*in vivo* top; *in vitro* bottom). (C) Mesenchyme trajectory (*in vivo* top; *in vitro* bottom). (D) First and Second Heart Field trajectories (*in vivo* only).

Development • Supplementary information

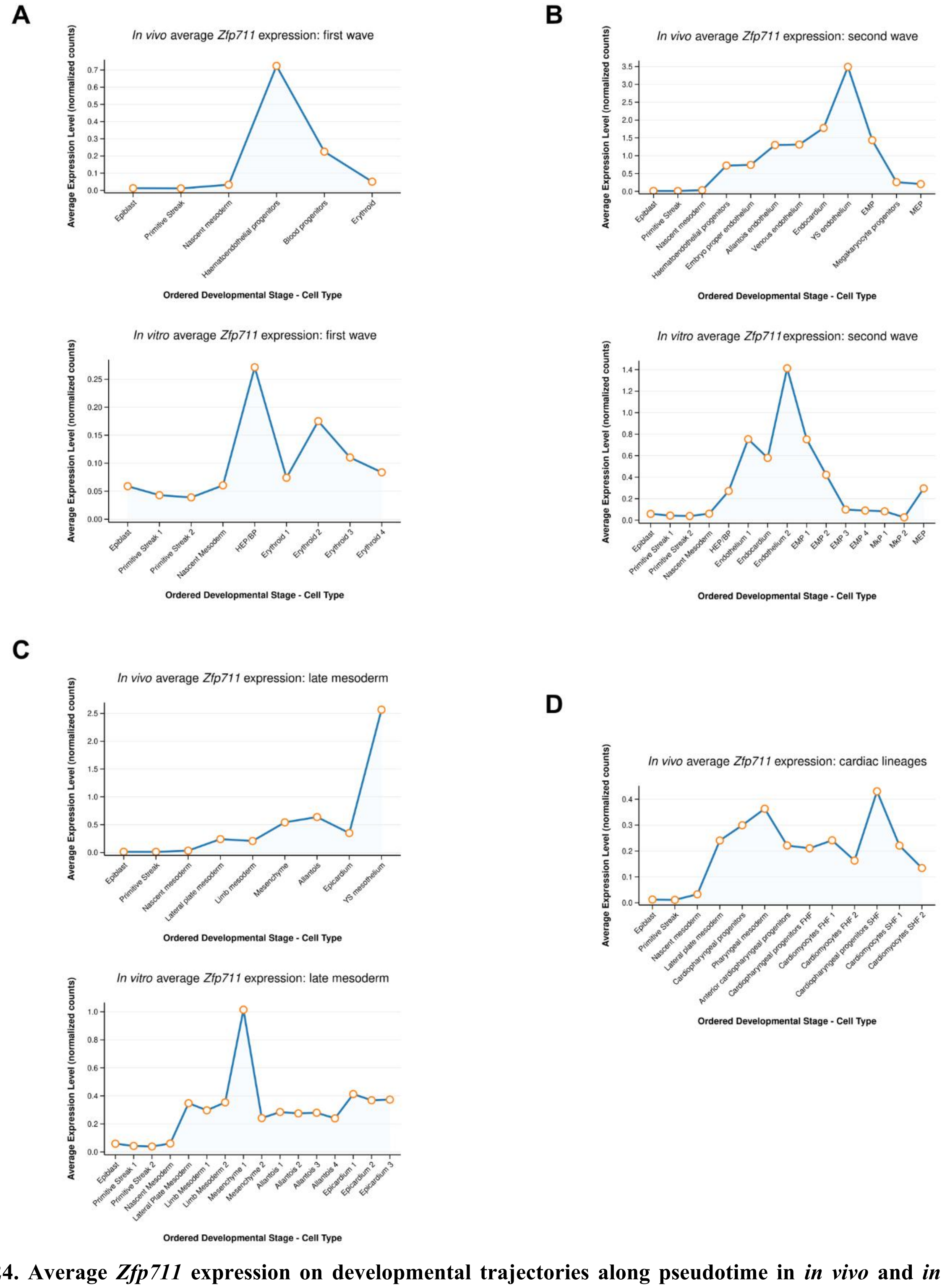

Fig. S24. Average *Zfp711* expression on developmental trajectories along pseudotime in *in vivo* and *in vitro* data (after manual ordering of cell types). (A) Hematopoietic primitive wave trajectory shown for *in vivo* (top) and *in vitro* (bottom) datasets. (B) Hematopoietic second wave trajectory (*in vivo* top; *in vitro* bottom). (C) Late mesoderm trajectory (*in vivo* top; *in vitro* bottom). (D) Cardiac trajectory (*in vivo* only).

Development • Supplementary information

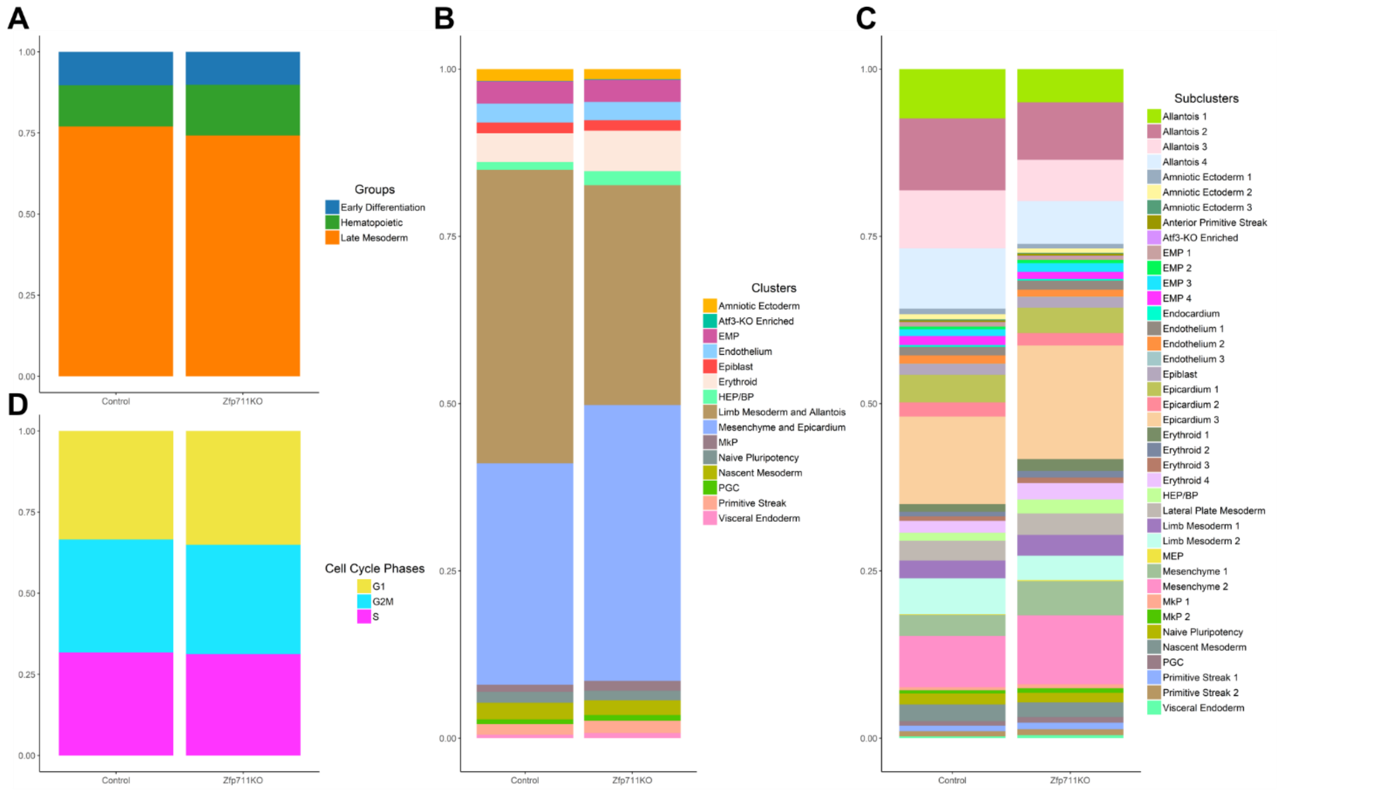

Fig. S25. Δ*Zfp711* Differential Abundance Analysis results with Speckle R Package. (A-D) Fraction of the annotated cell types in order of groups (A), clusters (B), sub-clusters (C), and cell cycle (D).

Development • Supplementary information

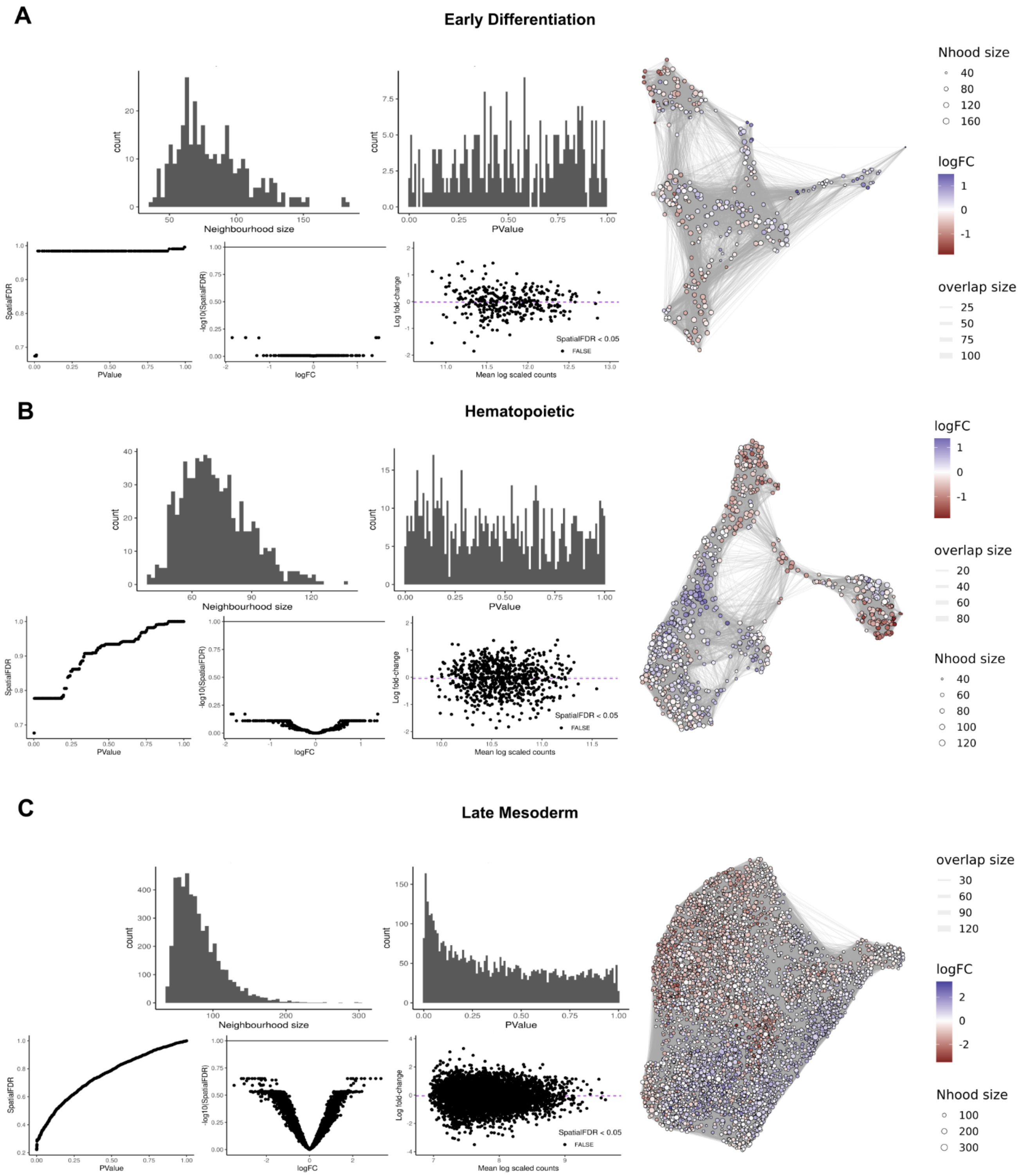

Fig. S26. Quality control and visualization of MiloR differential abundance analysis for *Zfp711*-KO. (A) Early Differentiation, (B) Hematopoietic, and (C) Late Mesoderm. For each comparison, the plots show: the neighborhood graph colored by log fold change (logFC), with node size scaled by cell count; histograms of neighborhood sizes and p-values; the relationship between p-values and the spatially-corrected FDR (SpatialFDR); a volcano plot (logFC vs. −log10(SpatialFDR)); and an MA plot (logFC vs. mean abundance). In relevant plots, significant neighborhoods (SpatialFDR < 0.05) are highlighted in red. UMAP with neighborhood overlay (node size = neighborhood size. Color scale indicates log fold-change (red = decreased, blue = increased in Δ*Zfp711*).

Development • Supplementary information

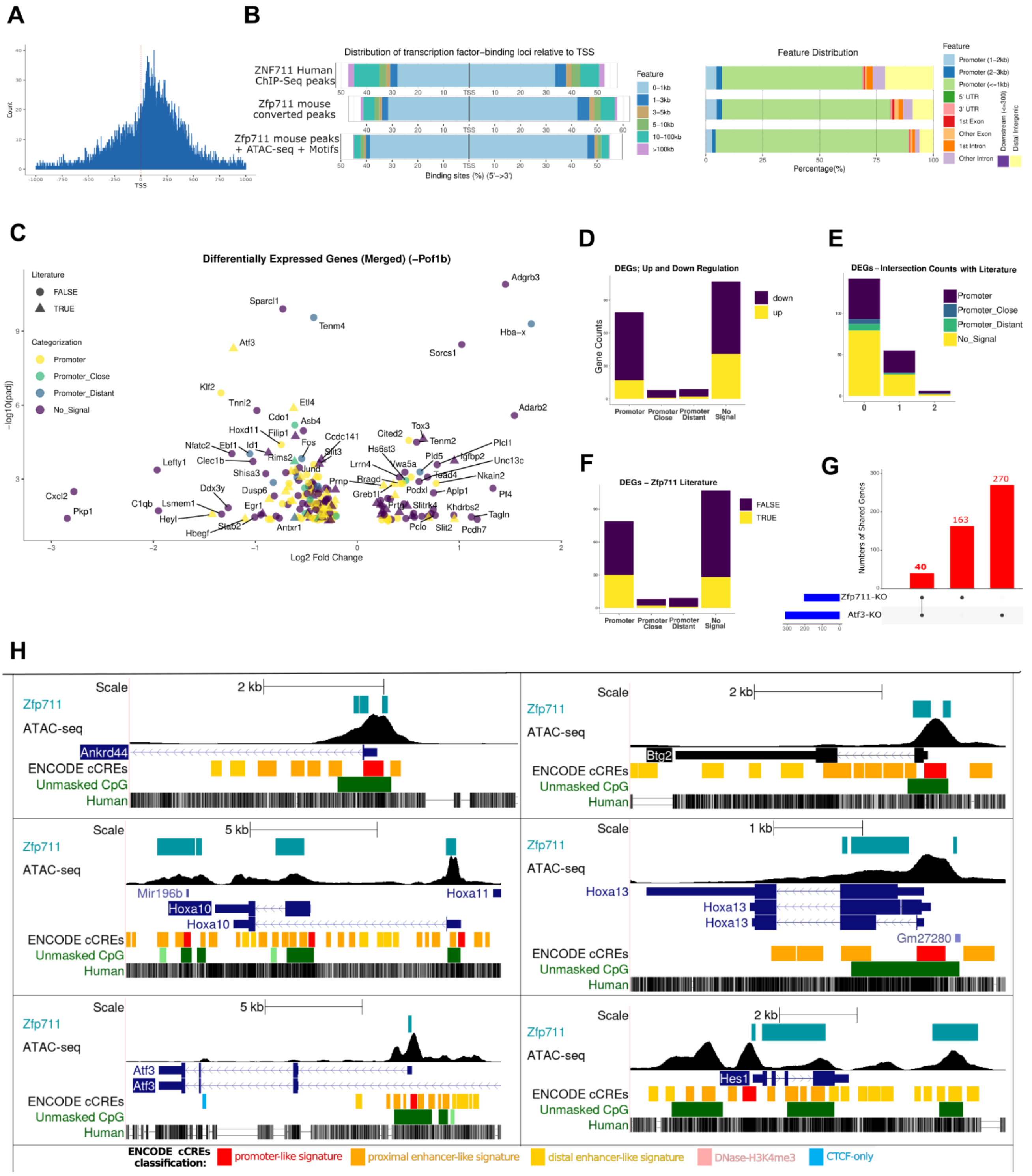

Fig. S27. Results and categorization of the Differential Gene Expression Analysis of the Δ*Zfp711* cells. (A) *Zfp711* binding regions distribution histogram relative to the Transcription Start Site (TSS). (B) Location and distance to the TSS of the *Zfp711* ChIP-seq peak regions in three stages of the analysis from human to mouse. (C) Volcano plot of the total significant DEGs. (D-G) Distribution of the DEGs, *Zfp711*-binding categorization, shared DEGs with Δ*Atf3* cells, and DEGs (as reported in the literature). (H) Some examples of *Zfp711* binding sites with ATAC-seq peaks in three example DEGs. Supporting data can be seen in Table S4L-S,S6B, and S7F-J.

Development • Supplementary information

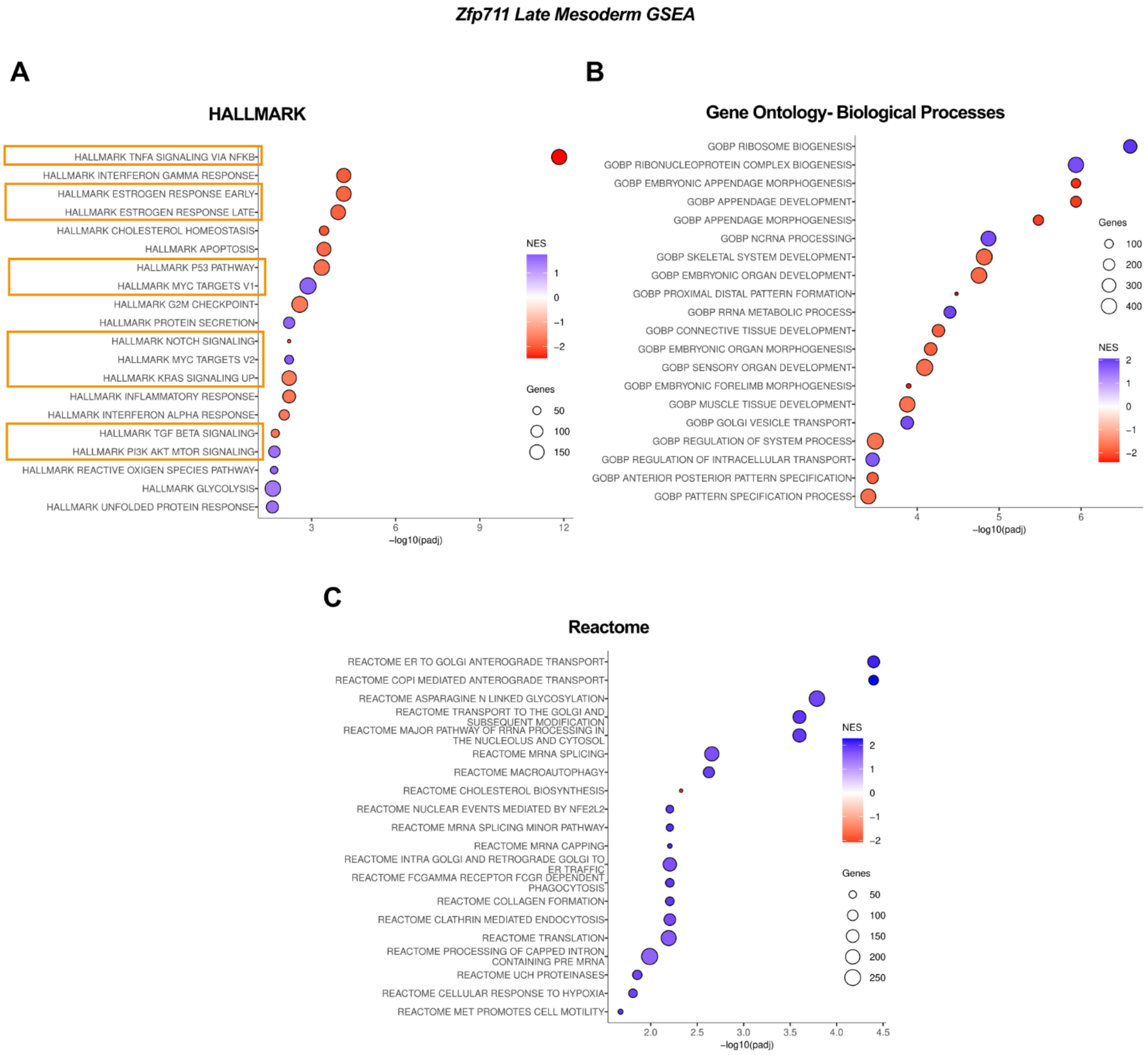

Fig. S28. GSEA Results of the Δ*Zfp711* cells as Late Mesoderm. (A-C) Show the significance (-log10 (adjusted-P)) of the GSEA for 3 collections of gene sets (from MSigDB, https://www.gsea-msigdb.org/) and the top-20 gene sets in that collection (on the left). The size of the circle indicates the number of genes in a gene set (after removal of the genes expressed at a very low level). Blue to red shading indicates the increase or decrease of the Normalized Enrichment Score (NES). The orange-colored boxes highlight "signaling pathways."

Development • Supplementary information

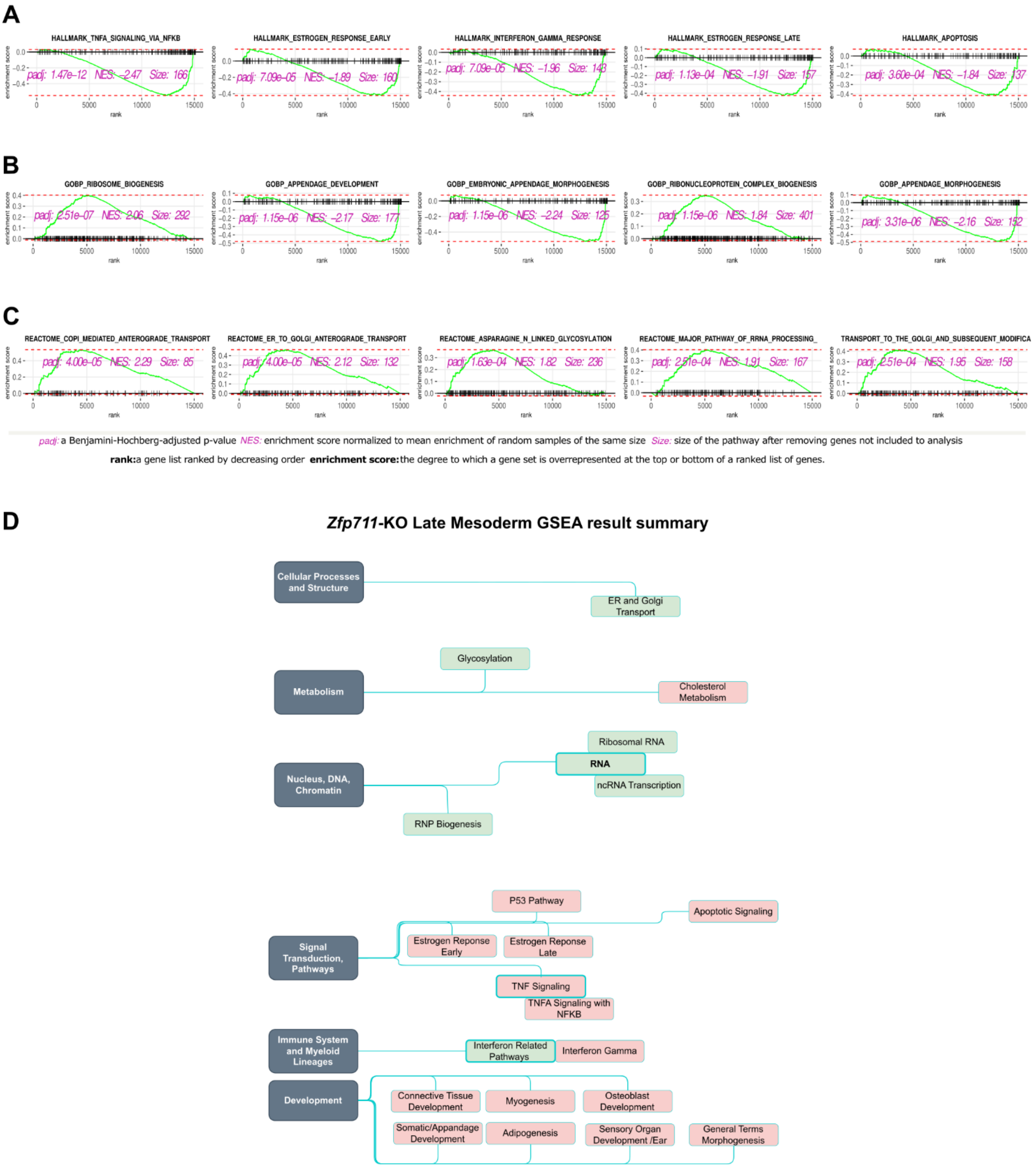

Fig. S29. GSEA results of the Δ*Zfp711* cells as Late Mesoderm. (A-C) Show enrichment plots of the top-5 results from Late Mesoderm GSEA results. (D) The manually grouped GSEA results of the Δ*Zfp711* cells in Late Mesoderm. Supporting data can be seen in Table S5G-I.

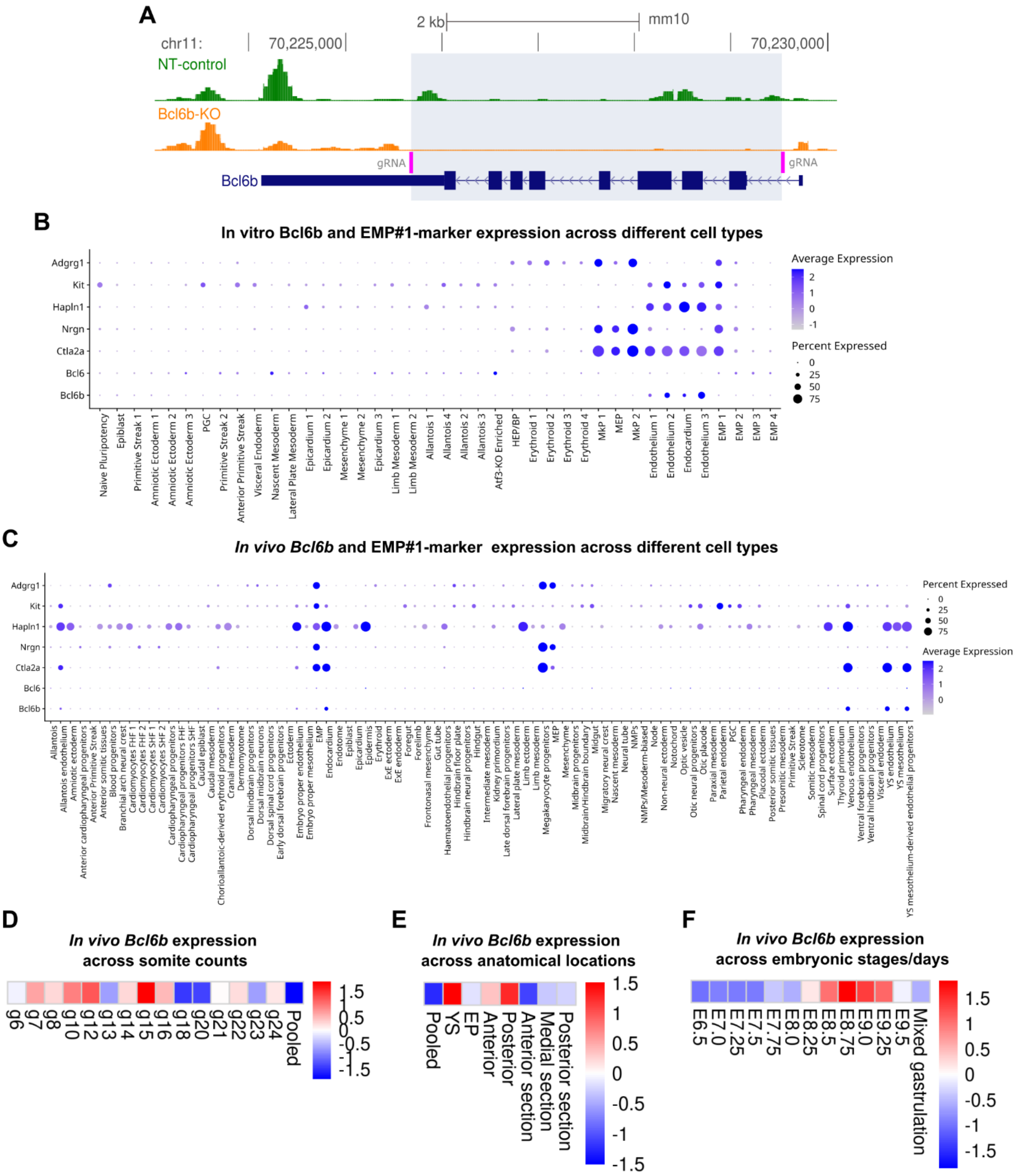

Fig. S30. *Bcl6b* locus targeting and expression dynamics across *in vitro* and *in vivo* datasets. (A) Genome-browser view of the *Bcl6b* locus. Expression reads are shown for a NT-control (green) and the *Bcl6b*-KO sample (red). Positions of the gRNAs used for the KO are indicated. (B) Dotplot of expression for *Bcl6b*, *Bcl6b*-paralog and EMP#1-marker genes across *in vitro* subclusters. (C) The same gene set profiled for all cell types *in vivo* from the reference atlas. (D–F) *In vivo Bcl6b* expression summarized across (D) somite stages/counts, (E) anatomical locations, and (F) embryonic days. For all heat maps, color indicates z-scored/normalized average expression per cell type or group (red = higher, blue = lower). Abbreviations: NT, non-targeting control; KO, knockout; YS, yolk sac; EP, embryo proper.

Development • Supplementary information

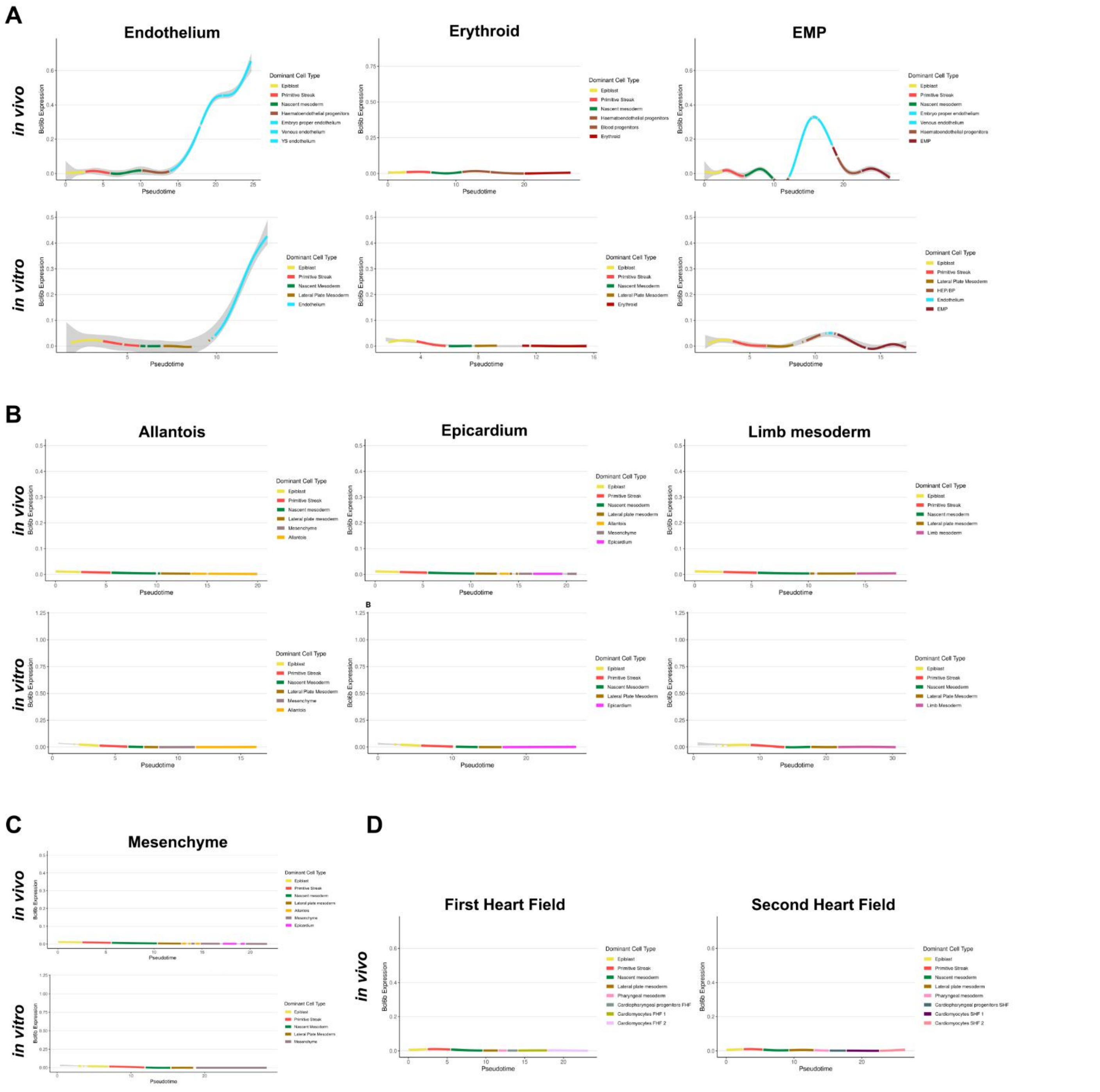

Fig. S31. *Bcl6b* expression on developmental trajectories along pseudotime in *in vivo* and *in vitro* data. (A–D) For each lineage, Slingshot was used to infer trajectories and compute pseudotime (x-axis). Curves show the loess-smoothed mean scaled expression (log-normalized) of lineage-specific marker sets (y-axis) along each trajectory. Shaded ribbons indicate the 95% confidence interval. Colored bars beneath each curve denote the dominant cell-type label at that pseudotime segment (legend at right). (A) Endothelium, Erythroid, and EMP trajectories shown for *in vivo* (top) and *in vitro* (bottom) datasets. (B) Allantois, Epicardium, and Limb mesoderm trajectories (*in vivo* top; *in vitro* bottom. (C) Mesenchyme trajectory (*in vivo* top; *in vitro* bottom). (D) First and Second Heart Field trajectories (*in vivo* only).

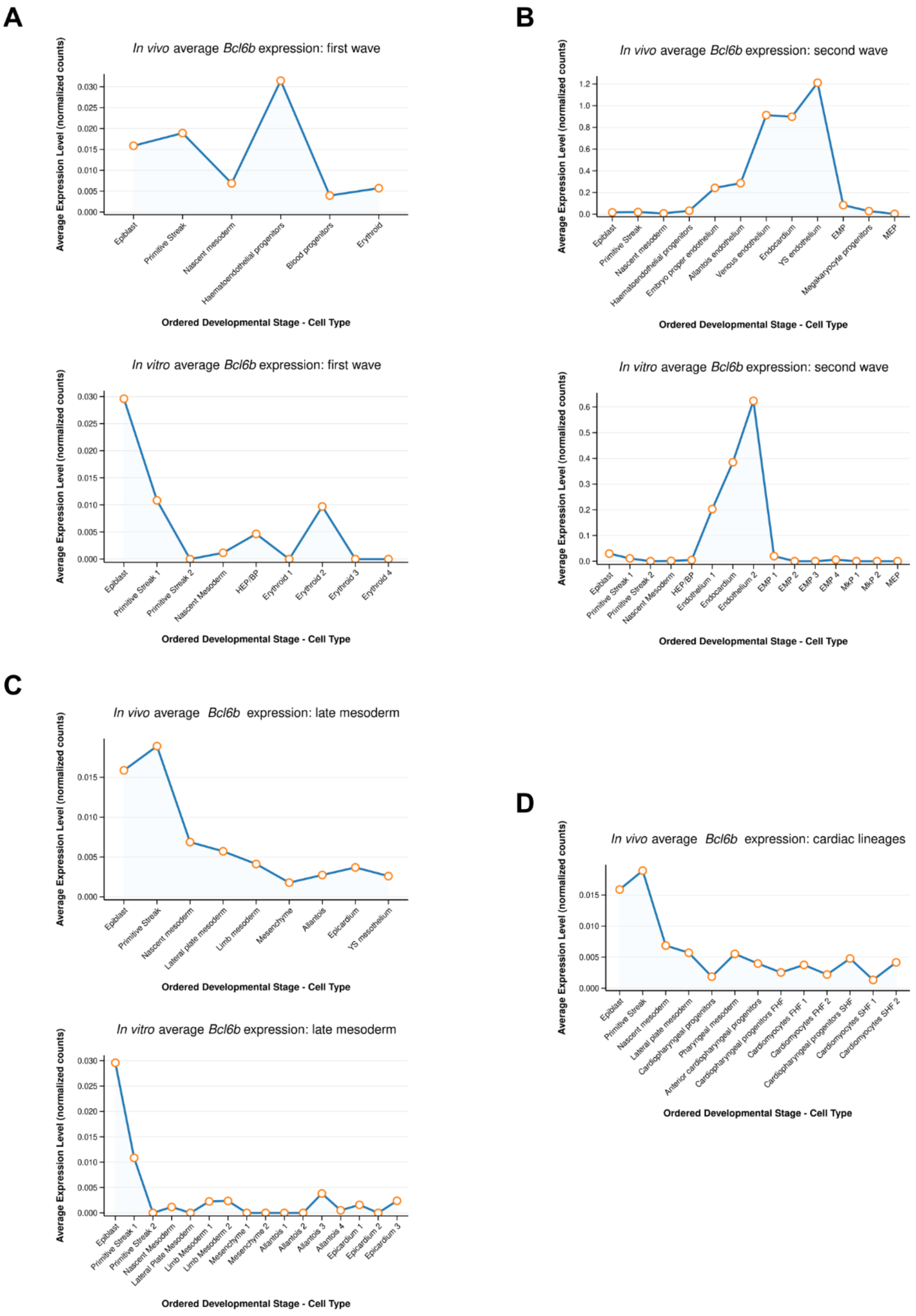

Fig. S32. Average *Bcl6b* expression on developmental trajectories along pseudotime in *in vivo* and *in vitro* data (after manual ordering of cell types). (A) Hematopoietic primitive wave trajectory shown for *in vivo* (top) and *in vitro* (bottom) datasets. (B) Hematopoietic second wave trajectory (*in vivo* top; *in vitro* bottom). (C) Late mesoderm trajectory (*in vivo* top; *in vitro* bottom). (D) Cardiac trajectory (*in vivo* only).

Development • Supplementary information

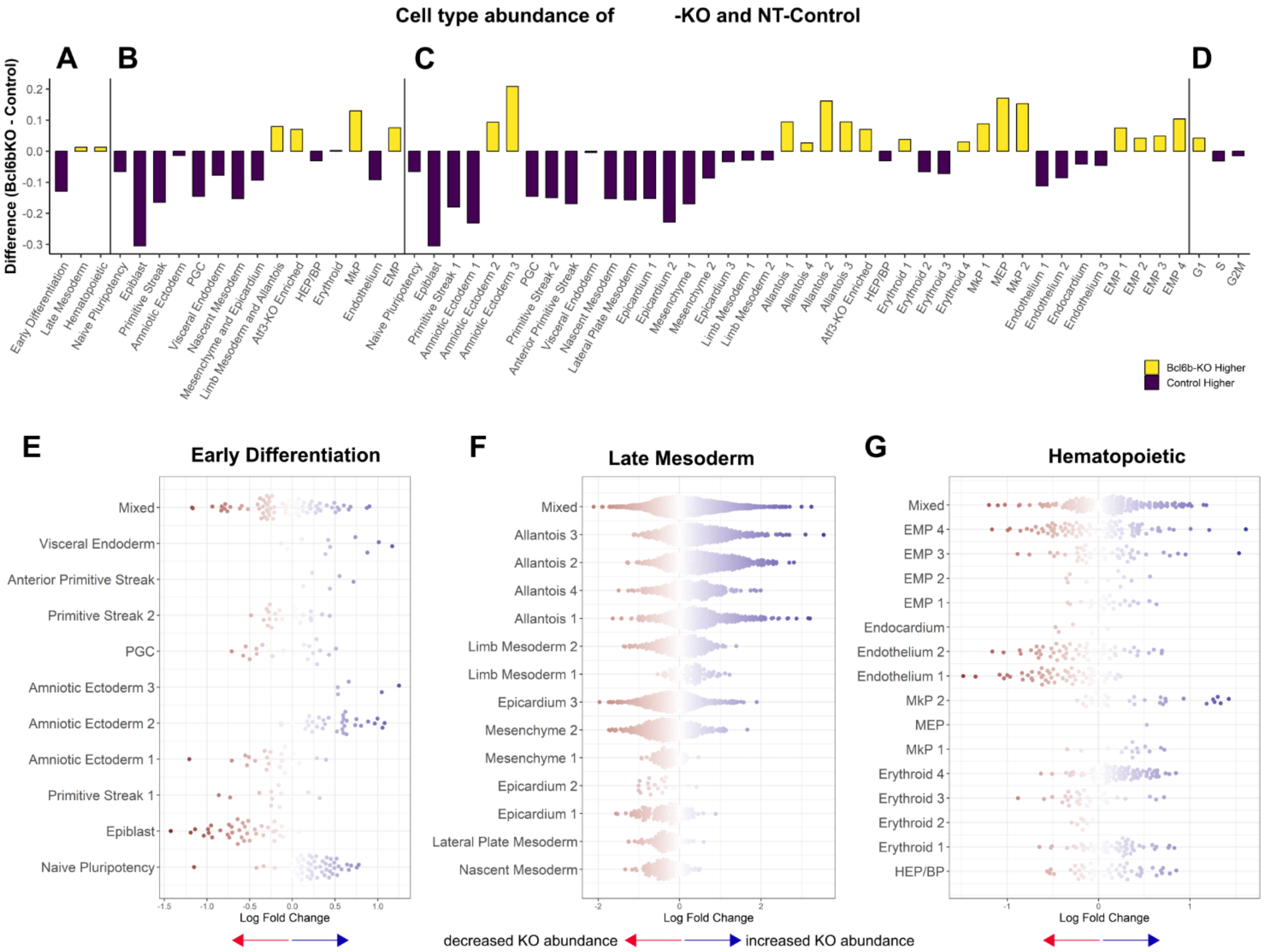

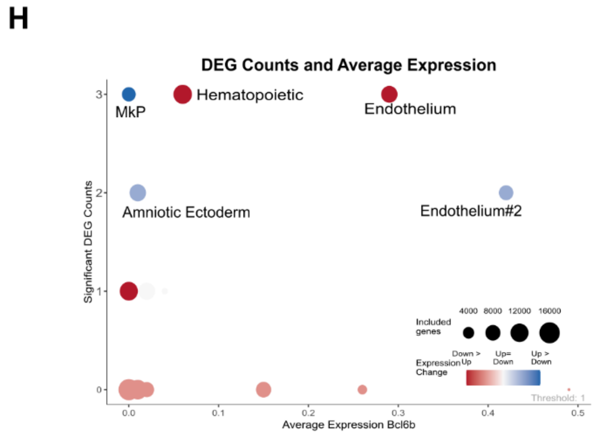

I

DEGs

Gene Name	baseMean	Clusters	Regulation
Bcl6b	44	Endothelium;Hematopoietic	down
Cryab	51	Amniotic Ectoderm#3	down
Dqx1	46	Allantois#3	down
Hba-a1	48	Endothelium	down
Pcdh17	223	Hematopoietic	down
Ptprm	21	Naive Pluripotency	down
Rhox5	23	Erythroid#1;Erythroid	down
Ccl12	82	EMP#4	up
Cdkn1c	9	Atf3-KO-Enriched	up
Col1a1	27	Amniotic Ectoderm	up
Col1a2	29	Amniotic Ectoderm	up
Ctla2a	1586	Megakaryocyte	up
Hba-x	250	Epiblast	up
Hist1h1c	89	MkP	up
Myl9	45	MkP	up
Nrk	111	MkP	up
Prkg1	14	MEP	up
Slc16a13	84	Endothelium#2;Endothelium;Hematopoietic	up

Fig. S33. Results with Δ*Bcl6b* cells. (A-C) Compare the number of NT-Control versus the Δ*Bcl6b* cells in groups, clusters, and sub-clusters. (D) compares the number of cells in the respective phases of the cell cycle. (E-G) Show the fold-change in the differential abundance analysis in the different groups while creating small neighborhoods (small groups of cells) shown as dots (each dot represents mainly 50-100 cells). The text on the y-axis indicates sub-clusters, and the log fold change is shown on the x-axis. "Mixed" on the y-axis indicates a neighborhood that does not belong to any sub-cluster (using a 50% threshold). (H) Shows the average expression level and significant DEGs (adjusted-P < 0.05) in groups, clusters, and sub-clusters. The size of the circles indicates the number of genes included in the analysis after removing the low-expressed genes in the group, cluster, or sub-cluster. The color of the circle indicates up- or downregulation (*blue* and *red*, respectively). (I) Shows all the DEGs, their expression level (*baseMean*), and to which cluster they belong.

Development • Supplementary information

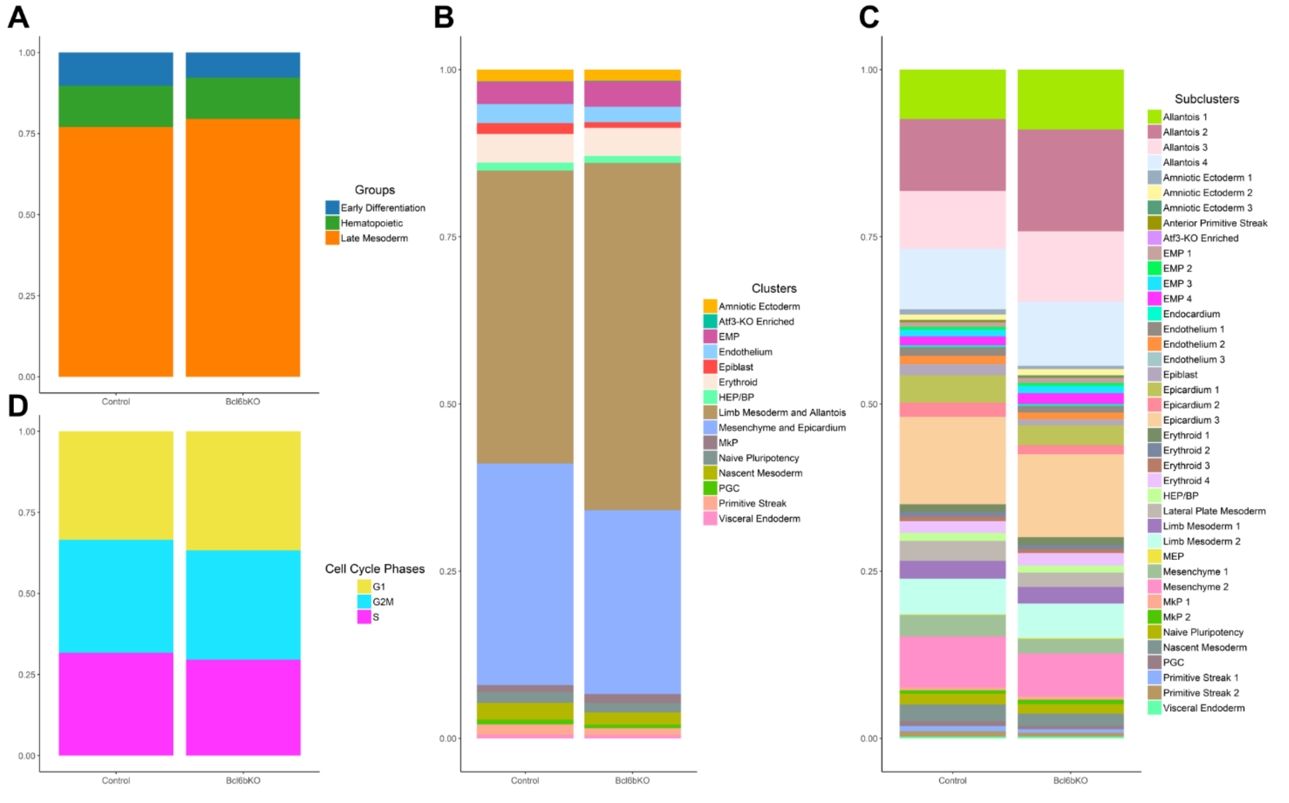

Fig. S34. Δ*Bcl6b* cells: Differential Abundance Analysis results with Speckle R Package. (A-D) Fraction of the annotated cell types in order of groups (A), clusters (B), sub-clusters (C), and cell cycle (D). Outputs of the Speckle differential abundance analysis can be seen in Table S2Q-X.

Development • Supplementary information

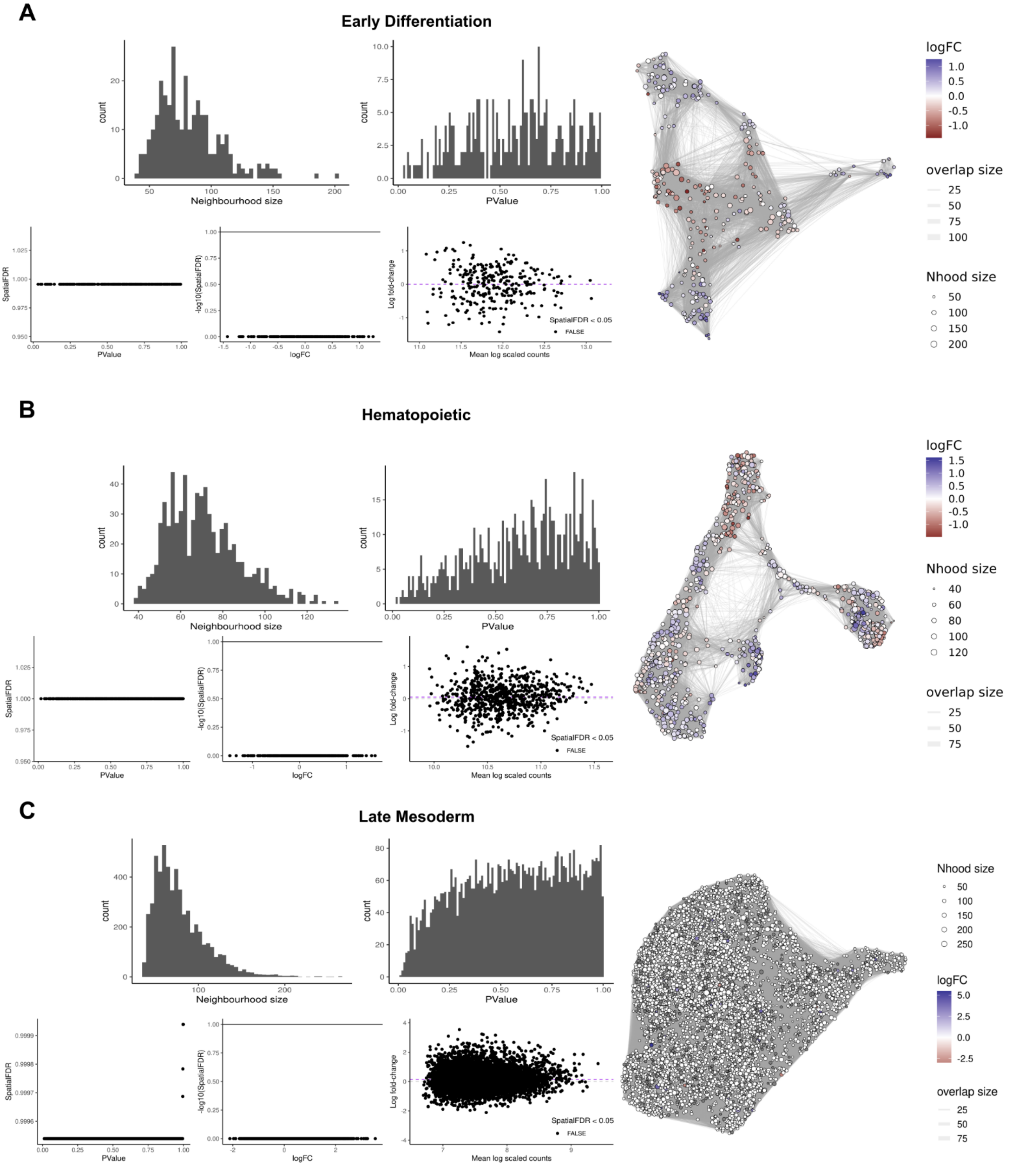

Fig. S35. Quality control and visualization of MiloR differential abundance analysis for *Bcl6b*-KO. (A) Early Differentiation , (B) Hematopoietic, and (C) Late Mesoderm group. For each comparison, the plots show: the neighborhood graph colored by log fold change (logFC), with node size scaled by cell count; histograms of neighborhood sizes and p-values; the relationship between p-values and the spatially-corrected FDR (SpatialFDR); a volcano plot (logFC vs. −log10(SpatialFDR)); and an MA plot (logFC vs. mean abundance). In relevant plots, significant neighborhoods (SpatialFDR < 0.05) are highlighted in red. UMAP with neighborhood overlay (neighborhood size. Color scale indicates log fold-change (red = decreased, blue = increased in Δ*Bcl6b*).

Development • Supplementary information

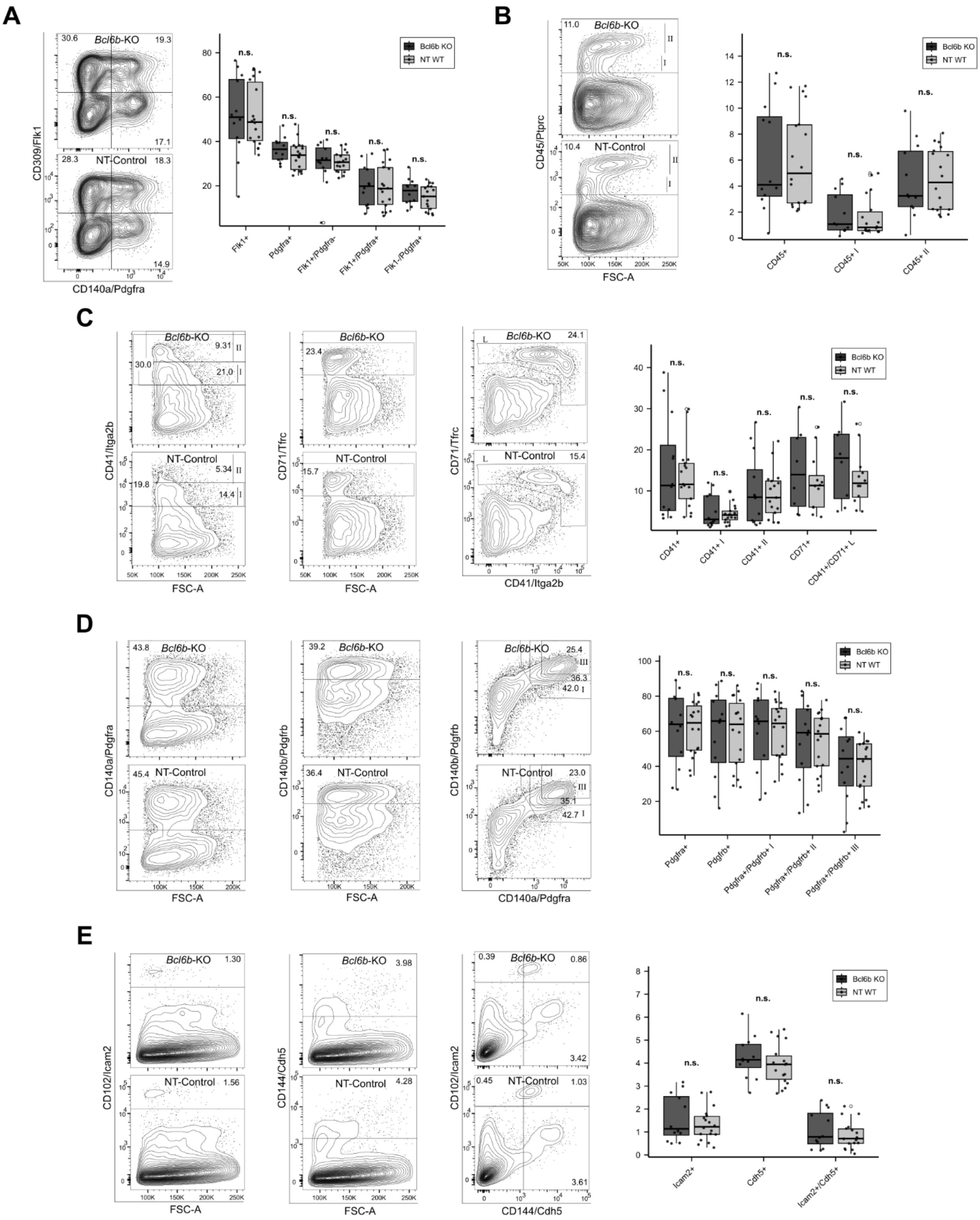

Fig. S36. Flow cytometric validation of *Bcl6b* knockout effects on mesodermal and hemato-endothelial differentiation. (A) D4 analysis showing distribution of hemato-endothelial progenitors (Flk1+/Pdgfra-) and mesodermal populations (Flk1+/Pdgfra+) in Δ*Bcl6b* versus NT-Control cells. Representative plots (left) and quantification (right). (B) D7 EMP analysis using CD45 marker. Representative plots and quantification in CD45+ EMPs in Δ*Bcl6b*. (C) D7 erythroid lineage assessment using CD41 and CD71 markers. (D) D7 late mesoderm populations marked by Pdgfra/Pdgfrb. (E) D7 analysis of endothelial markers. Representative plots and quantification in Cdh5+/Icam2+ endothelial cells in Δ*Bcl6b*. All data represent mean ± SEM from three independent experiments with 4 knockout and 6 control clones. Statistical significance: *p<0.05, **p<0.01, ***p<0.001; n.s., not significant.

Development • Supplementary information

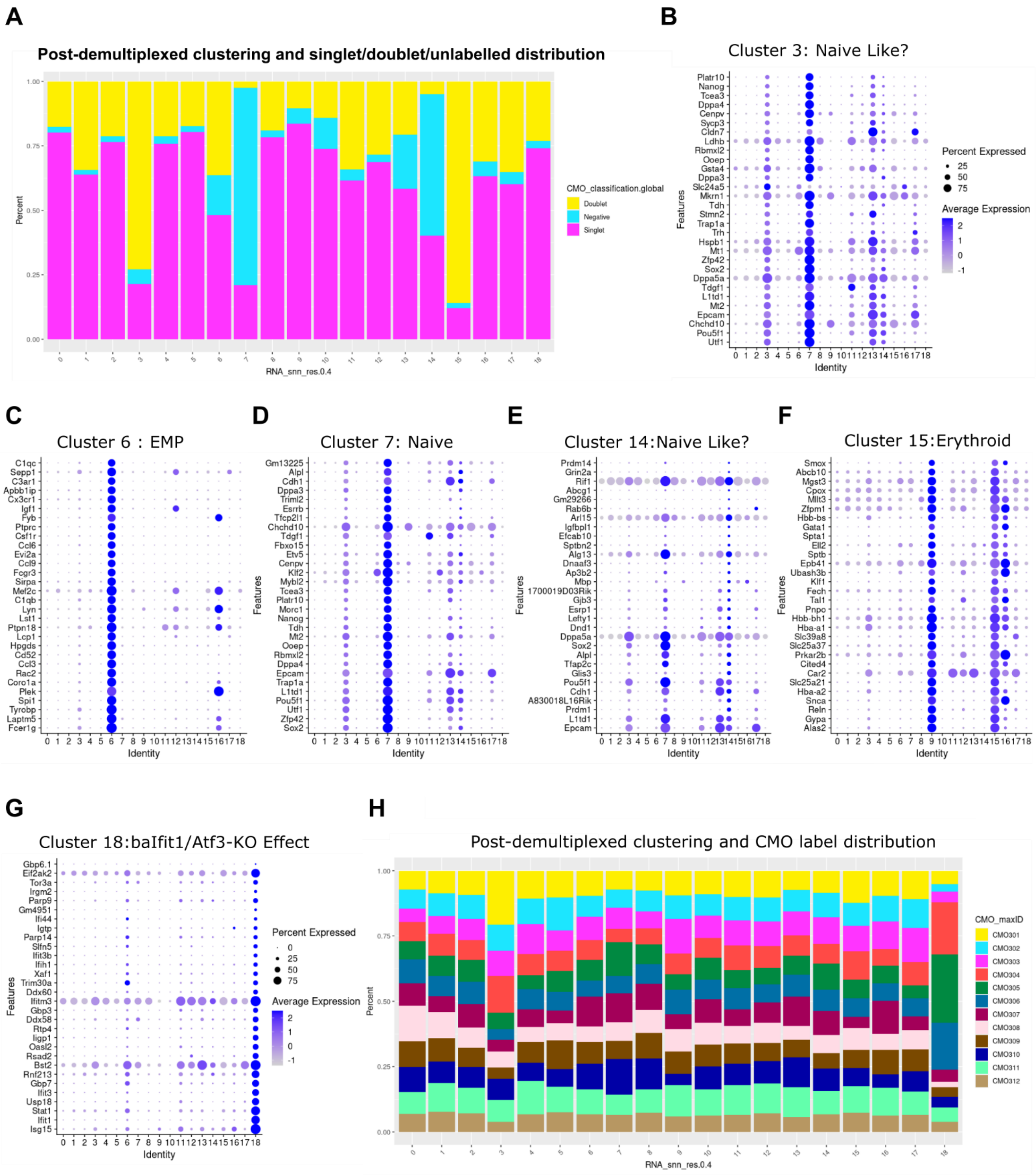

Fig. S37. Demultiplexing results of CMO labels and their distribution across cell types. (A) Distribution of demultiplexed CMO labels into doublets, singlets, and negatives (unlabeled) following quality control steps. The X-axis represents clusters (0-18), and the Y-axis shows the percentage distribution of doublets, singlets, and negatives. (B-F) A plot of the highly expressed genes in the clusters identified in panel A. (G) Displays the highly expressed genes in Cluster 18. (H) Shows the distribution of CMO labels across clusters.

Development • Supplementary information

Table S1. Flow Cytometric Analysis Data and Statistical Test Results.

Supplementary Table_1A Atf3-KO_FCA_mixed_effects Atf3 KOvsNT ControlResults

Supplementary Table_1B Atf3-KO_FCA_freq_dataAtf3 KOvsNT ControlDataset

Supplementary Table_1C Zfp711-KO_FCA_mixed_effects Zfp711 KOvsNTControlResults

Supplementary Table_1D Zfp711-KO_FCA_freq_data Zfp711 KOvsNT ControlDataset

Supplementary Table_1E Bcl6b-KO_FCA_mixed_effectsBcl6b KOvsNT ControlResults

Supplementary Table_1F Bcl6b-KO_FCA_freq_data Bcl6b KOvsNT ControlDataset

The supplementary data for flow cytometric analysis includes results from cell surface marker staining at Day 4 and Day 7. Panels A, C, and E present the mixed-effects statistical analysis, while Panels B, D, and F show the frequency data for the staining panels.

Available for download at
https://journals.biologists.com/dev/article-lookup/doi/10.1242/dev.204792#supplementary-data

Table S2. Differential Abundance Analysis results using Speckle (DAA-Speckle)

Supplementary Table 2A.DAA-Speckle-asin Results.Atf3-KO vs NT-Control. Groups.

Supplementary Table 2B.DAA-Speckle-asin Results.Atf3-KO vs NT-Control. Clusters.

Supplementary Table 2C.DAA-Speckle-asin Results.Atf3-KO vs NT-Control. Sublusters.

Supplementary Table 2D.DAA-Speckle-asin Results.Atf3-KO vs NT-Control. Cell Cycle.

Supplementary Table 2E.DAA-Speckle-logit Results.Atf3-KO vs NT-Control. Groups.

Supplementary Table 2F.DAA-Speckle-logit Results.Atf3-KO vs NT-Control. Clusters.

Supplementary Table 2G.DAA-Speckle-logit Results.Atf3-KO vs NT-Control. Sublusters.

Supplementary Table 2H.DAA-Speckle-logit Results.Atf3-KO vs NT-Control. Cell Cycle.

Supplementary Table 2I.DAA-Speckle-asin Results.Zfp711-KO vs NT-Control. Groups.

Supplementary Table 2J.DAA-Speckle-asin Results.Zfp711-KO vs NT-Control. Clusters.

Supplementary Table 2K.DAA-Speckle-asin Results.Zfp711-KO vs NT-Control. Sublusters.

Supplementary Table 2L.DAA-Speckle-asin Results.Zfp711-KO vs NT-Control. Cell Cycle.

Supplementary Table 2M.DAA-Speckle-logit Results.Zfp711-KO vs NT-Control. Groups.

Supplementary Table 2N.DAA-Speckle-logit Results.Zfp711-KO vs NT-Control. Clusters.

Supplementary Table 2O.DAA-Speckle-logit Results.Atf3-KO vs NT-Control. Sublusters.

Supplementary Table 2P.DAA-Speckle-logit Results.Zfp711-KO vs NT-Control. Cell Cycle.

Supplementary Table 2Q.DAA-Speckle-asin Results.Bcl6b-KO vs NT-Control. Groups.

Supplementary Table 2R.DAA-Speckle-asin Results.Bcl6b-KO vs NT-Control. Clusters.

Supplementary Table 2S.DAA-Speckle-asin Results.Bcl6b-KO vs NT-Control. Subclusters.

Supplementary Table 2T.DAA-Speckle-asin Results.Bcl6b-KO vs NT-Control. Cell Cyle.

Supplementary Table 2U.DAA-Speckle-logit Results.Bcl6b-KO vs NT-Control. Groups.

Supplementary Table 2V.DAA-Speckle-logit Results.Bcl6b-KO vs NT-Control. Clusters.

Supplementary Table 2W.DAA-Speckle-logit Results.Bcl6b-KO vs NT-Control. Subclusters.

Supplementary Table 2X.DAA-Speckle-logit Results.Bcl6b-KO vs NT-Control. Cell Cycle.

Results from the Speckle differential abundance test (using t-test) at the group, cluster, and subcluster levels, with transformations applied using the arcsine square root transformation ('asin') and logit transformation ('logit').

Available for download at
https://journals.biologists.com/dev/article-lookup/doi/10.1242/dev.204792#supplementary-data

Table S3. Differential Abundance Analysis Results using miloR (DAA-miloR).

sTable_3A_Atf3_Early_Differentiation Atf3 KO vs NT Control

sTable_3B_Atf3_Late_Mesoderm Atf3 KO vs NT Control
sTable_3C_Atf3_Hematopoietic Atf3 KO vs NT Control

sTable_3D_Zfp711_Early_Differen Zfp711 KO vs NT Control

sTable_3E_Zfp711_Late_Mesoderm Zfp711 KO vs NT Control

sTable_3F_Zfp711_Hematopoietic Zfp711 KO vs NT Control

sTable_3G_Bcl6b_Early_Differentiation Bcl6b KO vs NT Control

sTable_3H_Bcl6b_Late_Mesoderm Bcl6b KO vs NT Control

sTable_3I_Bcl6b_Hematopoietic Bcl6b KO vs NT Control

The results of the miloR differential abundance analysis that seperated by groups and conditions.

Available for download at
https://journals.biologists.com/dev/article-lookup/doi/10.1242/dev.204792#supplementary-data

Table S4. Differential Gene Expression Analysis (DGEA) Results.

Supplementary Table_4A_Atf3_total_DEGs_list; DESeq2 Results (Combined) and Intersections with ChIP-Peaks, Literature DEGs, ENCODE TF Targets

Supplementary Table_4B_Atf3_DESeq2_summary; DESeq2 Summary

Supplementary Table_4C_Atf3_DESeq2_Late_Mesoderm; DESeq2 Results (padj < 0.1)

Supplementary Table_4D_Atf3_DESeq2_Hematopoietic; DESeq2 Results (padj < 0.1)

Supplementary Table_4E_Atf3_DESeq2_EMP; DESeq2 Results (padj < 0.1)

Supplementary Table_4F_Atf3_DESeq2_Hoxa_Mesoderm; DESeq2 Results (padj < 0.1)

Supplementary Table_4G_Atf3_DESeq2_Hoxb_Mesoderm; DESeq2 Results (padj < 0.1)

Supplementary Table_4H_Atf3_DESeq2_EMPCcl4Sepp1; DESeq2 Results (padj < 0.1)

Supplementary Table_4I_uniq_baIfit1; markers for baIfit1 (padj < 0.1)

Supplementary Table_4J_uniq_EndRsad2; markers for EndRsad2 (padj < 0.1)

Supplementary Table_4K_uniq_EndCyp26b1; markers for EndCyp26b1 (padj < 0.1)

Supplementary Table_4L_Zfp711_total_DEGs_list; DESeq2 Results (Combined) and Intersections with ChIP-Peaks, Literature DEGs, ENCODE TF Targets

Supplementary Table_4M_Zfp711_DESeq2_summary; DESeq2 Summary

Supplementary Table_4N_Zfp711_DESeq2_Late_Mesoderm; DESeq2 Results (padj < 0.1)

Supplementary Table_4O_Zfp711_DESeq2_Hematopoietic; DESeq2 Results (padj < 0.1)

Supplementary Table_4Q_Zfp711_DESeq2_Hoxa_Mesoderm; DESeq2 Results (padj < 0.1)

Supplementary Table_4P_Zfp711_DESeq2_Hoxb_Mesoderm; DESeq2 Results (padj < 0.1)

Supplementary Table_4R_Zfp711_DESeq2_Endothelium; DESeq2 Results (padj < 0.1)

Supplementary Table_4S_Zfp711_DESeq2_aFgf12SG2M; DESeq2 Results (padj < 0.1)

Supplementary Table_4T_Zfp711_DESeq2_bPlxna4; DESeq2 Results (padj < 0.1)

Supplementary Table_4U_Bcl6b_total_DEGs_list; DESeq2 Results (Combined) and Intersections with ChIP-Peaks, Literature DEGs, ENCODE TF Targets

Supplementary Table_4V_Bcl6b_DESeq2_summary; DESeq2 Summary

Supplementary Data of the Differential Gene Expression Analysis.

Available for download at
https://journals.biologists.com/dev/article-lookup/doi/10.1242/dev.204792#supplementary-data

Development • Supplementary information

Table S5. Gene Set Enrichment Analysis Results.(GSEA).

Supplementary Table_5A_Atf3_EMP_HALLMARK

Supplementary Table_5B_Atf3_EMP_GOBP

Supplementary Table_5C_Atf3_EMP_REACTOME

Supplementary Table_5D_Atf3_Late_Mesoderm_HALLMARK

Supplementary Table_5E_Atf3_Late_Mesoderm_GOBP

Supplementary Table_5F_Atf3_Late_Mesoderm_REACTOME

Supplementary Table_5G_Zfp711_Late_Mesoderm_HALLMARK

Supplementary Table_5H_Zfp711_Late_Mesoderm_GOBP

Supplementary Table_5I_Zfp711_Late_Mesoderm_REACTOME

Available for download at
https://journals.biologists.com/dev/article-lookup/doi/10.1242/dev.204792#supplementary-data

Fig. S6. Differentially expressed genes (DEGs) from Atf3, Zfp711, and Bcl6b knockout and knockdown studies, converted to mouse orthologs.

Supplementary Table_6A_Atf3_Literature_DEGs

Supplementary Table_6B_Zfp711_Literature_DEG

Supplementary Table_6C_Atf3_TF_Targets_Harmonize_mouse_converted

Available for download at
https://journals.biologists.com/dev/article-lookup/doi/10.1242/dev.204792#supplementary-data

Table S7. ChIP-seq coordinates and GREAT peak-to-gene links.

Supplementary Table_7A_ATF3_Human_hg38_ChIP_combined

Supplementary Table_7B_Atf3_Mouse_mm10_ChIP_converted

Supplementary Table_7C_Atf3_Mouse_mm10_ChIP_intersected

Supplementary Table_7D_Atf3_GREAT_all_genes_mm10

Supplementary Table_7E_Atf3_GREAT_all_region_mm10

Supplementary Table_7F_ZNF711_Human_hg38_ChIP_combined

Supplementary Table_7G_Zfp711_Mouse_mm10_ChIP_converted

Supplementary Table_7H_Zfp711_Mouse_mm10_ChIP_intersected

Supplementary Table_7I_Zfp711_GREAT_all_gene_mm10

Supplementary Table_7J_Zfp711_GREAT_all_region_mm10

Available for download at
https://journals.biologists.com/dev/article-lookup/doi/10.1242/dev.204792#supplementary-data

Table S8. Data sets used in this study.

Supplementary Table_8A_Atf3_used_datasets

Supplementary Table_8B_Zfp711_used_datasets

Available for download at
https://journals.biologists.com/dev/article-lookup/doi/10.1242/dev.204792#supplementary-data

Table S9. Average Expression of Transcription Factors Atf3, Zfp711 and Bcl6b on groups, clusters and subclusters. (Combined scRNA-seq data (all KOs and NT-Control) and only NT-Control)

Supplementary Table9A_Atf3_Avg_Expr

Supplementary Table9B_Zfp711_Avg_Expr

Supplementary Table9C_Bcl6b_Avg_Expr

Available for download at
https://journals.biologists.com/dev/article-lookup/doi/10.1242/dev.204792#supplementary-data

Table S10. Selected DEGs for specific cell types and a complete list of DEGs with padj < 0.1.

Supplementary Table 10A. Atf3 Selected DEGs. (log2FC < -0.25   log2FC > 0.25)

Supplementary Table 10B. Atf3 DEGs padj < 0.1.

Supplementary Table 10C. Zfp711 Selected DEGs. (log2FC < -0.25 log2FC > 0.25)

Supplementary Table 10D. Zfp711 DEGs padj < 0.1.

Available for download at
https://journals.biologists.com/dev/article-lookup/doi/10.1242/dev.204792#supplementary-data

Development • Supplementary information
